# A recurrently perturbed 12-gene program marks drug-tolerant persister states across cancer types

**DOI:** 10.3389/fbinf.2026.1882238

**Published:** 2026-08-18

**Authors:** Itika Arora, Ahmed Mohammed Adam, Abdullah O. Aljaylani, Jumana Z. Alzuhayri, Ehab M. M. Ali, Faisal A. Alzahrani, Mohammad Imran Khan, Ahmed Yaqinuddin

**Affiliations:** 1 College of Medicine, Alfaisal University, Riyadh, Saudi Arabia; 2 Department of Biochemistry, Faculty of Science, King Abdulaziz University, Jeddah, Saudi Arabia; 3 Research Center, King Faisal Specialist Hospital and Research Center, Jeddah, Saudi Arabia

**Keywords:** cancer drug resistance, clinical utility, drug-tolerant persister cells, ferroptosis, gene expression profiling

## Abstract

Drug-tolerant persister (DTP) cells survive therapeutic stress through reversible, non-genetic adaptations, driving treatment failure across cancers. Whether a recurrently perturbed program defines the DTP state across cancer types remains poorly characterized. We performed integrated transcriptomic analysis across five GEO RNA-seq datasets representing breast, lung, pancreatic, colorectal, and melanoma DTP models. A stringent 12-gene signature, PanDTP-12, was established from genes meeting differential expression criteria (FDR <0.001, |log_2_FC| >= 0.8) across four discovery datasets. Module scores showed complete separation between persister and control samples (within-dataset and pooled AUC = 1.0; n = 16), though trivially small per-dataset sample sizes (n = 2–3 per group) preclude interpreting AUC = 1.0 as a stable performance estimate. Sensitivity analysis confirmed that all 12 genes remained recurrent when the fasting-quiescence dataset GSE214537 was excluded. In an independent PC9 NSCLC dataset (GSE255958), PanDTP-12 showed high directional concordance (9/12 genes) and quantitative module-score separation between parental PC9D and day-9 persister PC9O9 samples (AUC = 1.0, Cohen’s d = 14.76), though these metrics are based on only 6 samples and should be interpreted with caution. INK128-induced DTP models in MCF-7 and HCT116 cells demonstrated cell-cycle arrest, morphological changes, and RT-qPCR confirmation of PanDTP-12 upregulation (12/12 significant in HCT116; 6/12 in MCF-7). Drug-repurposing analysis identified focused drug-gene interactions among PanDTP-12 members. These findings identify PanDTP-12 as a recurrently perturbed twelve-gene program marking the DTP state across cancer types. The signature offers focused pharmacological hypotheses through druggable members (CLK1, XBP1, and KLF5) and establishes a framework for translational assessment of DTP-directed therapeutics, though larger validation cohorts and functional studies will be needed to confirm clinical utility.

## 1 Introduction

Drug resistance remains a central challenge in oncology, driving disease progression and treatment failure despite advances in targeted therapies and immunotherapy (Roszkowska, 2024; Tian et al., 2024). Beyond classical genetic mechanisms of resistance, both intrinsic and acquired mutations (Khan et al., 2024; Wang et al., 2019; Labrie et al., 2022), recent studies have revealed alternative non-genetic strategies by which cancer cells evade therapy, including epigenomic remodeling, transcriptional plasticity, and metabolic reprogramming (Marine et al., 2020; Bell and Gilan, 2020). Among these, drug-tolerant persister cells (DTPs) have emerged as a key non-genetic mechanism of resistance. DTPs represent a subpopulation of tumor cells that reversibly survive therapeutic stress and enter a quiescent, slow-proliferating state without acquiring *de novo* mutations (Laithy et al., 2024; Mikubo et al., 2021). Thus, temporary drug tolerance in these cells shares several key features and underlying mechanisms with embryonic diapause, a highly conserved developmental process in response to stress that halts cell division to ensure organismal survival (Rehman et al., 2021; Dhimolea et al., 2021). Specifically, DTPs exhibit the following characteristic features: (i) quiescence within cell cycles and mostly occurring in G1/G0 phases of these cycles (thus, ceasing the process of active proliferation), (ii) phenotypic plasticity and their capability of transitioning back into active tumor proliferative states after cessation of drug treatment, and (iii) recovery of their drug tolerance upon re-stimulation with drugs (Liau et al., 2017). Various converging mechanisms sustain DTP, including inhibition of apoptosis, metabolic reprogramming toward oxidative phosphorylation, epithelial-to-mesenchymal transformation, and substantial epigenetic modifications (Dhanyamraju et al., 2022). The landmark study by Sharma (Sharma et al., 2010) demonstrated the existence of a reversible, chromatin-driven, and drug-resistant condition in a subpopulation of cancer cells, thereby paving the way for DTP research. The combination of these mechanisms marks DTP cells as the originators of residual disease and potential relapse.

A detailed integrative transcriptome strategy was implemented in this work to address these limitations. We analyzed five publicly available RNA-seq datasets spanning distinct cancer types and DTP-inducing contexts through a stringent recurrence-based framework, performing differential expression within each dataset and then ranking genes by cross-dataset recurrence to minimize batch-driven artifacts. This approach yielded a stringent, recurrently perturbed 12-gene signature, PanDTP-12, which was evaluated for within- and cross-dataset discrimination, sensitivity to exclusion of the fasting-induced quiescence model, concordance-stratified sub-analysis (Core versus Variable subsets), quantitative external validation in an independent PC9 osimertinib dataset, and experimental confirmation by RT-qPCR in INK128-induced MCF-7 and HCT116 DTP models. Finally, recurrently perturbed genes, including PanDTP-12 members, were interrogated against pharmacogenomic resources to identify focused drug–gene interactions and exploratory hypotheses related to ferroptosis that could guide future DTP-targeted therapeutic strategies.

## 2 Materials and methods

### 2.1 Dataset selection and study design

Transcriptome data were sourced from the GEO database at the NCBI (Barrett et al., 2013). The selected datasets were used to demonstrate transcriptomic alterations in cancer persister cells across diverse cancers and therapeutic contexts by identifying DEGs that may contribute to the emergence of drug tolerance. All selected GEO series contained RNA-seq experiments (high-throughput sequencing-based expression profiling) performed on Illumina sequencing platforms. The datasets used for the analysis include: GSE84896 (BT474 breast cancer cells; screening dataset used only for initial DEG identification and not for PanDTP-12 derivation; parental cells vs. lapatinib-induced persister cells; gene-level FPKM output from CuffDiff), GSE153183 (PC9 non-small cell lung cancer cells; DMSO-treated control cells vs. osimertinib-induced DTP; FPKM matrix; n = 2 per group), GSE189764 (MIA PaCa-2 pancreatic cancer cells; control cells vs. Torin1/gemcitabine-induced persister cells; aggregation of transcript-level counts to gene-level TPM; n = 3 per group), and GSE214537 (RKO colorectal cancer cells; standard culture vs. nutrient restriction/fasting-induced quiescence; processed expression matrix; n = 3 per group). While GSE214537 represents a stress-induced quiescent state model rather than a pharmacologically induced drug-tolerability model, the fasting-induced quiescent state shares many similarities with drug-induced persister cells, including reversible proliferation arrest at the G1/G0 phase (Rehman et al., 2021).

This addition broadens the biological significance of the signature beyond drug-specific reactions. GSE246690 (melanoma cells; comparison of proliferating cells *vs* INK128-induced diapause-like DTP condition; count matrix; n = 4 per group). An independent dataset, GSE255958 (PC9 NSCLC cells; parental versus osimertinib day-9 persisters; RSEM gene-level TPM), was allocated for external validation and therefore not to generate the signature (Table 1). For quantitative external validation, parental PC9D samples were compared with osimertinib day-9 persister PC9O9 samples, reflecting the day-9 DTP state as defined in the GEO metadata and revised sample annotations.

**TABLE 1:** Summary of publicly available RNA-seq datasets from the Gene Expression Omnibus (GEO) used for discovery and external validation of the PanDTP-12 signature.

GEO accession	Cancer type	Cell line	Treatment	DTP/Ctrl	Format	DE method	Role
GSE153183	NSCLC	PC9	Osimertinib	2/2	FPKM	Limma	Discovery
GSE214537	Colorectal	RKO	Nutrient restriction	3/3	Counts	Limma	Discovery
GSE189764	Pancreatic	MIA PaCa-2	Torin1 + gemcitabine	3/3	TPM	Limma	Discovery
GSE246690	Melanoma	Melanoma	INK128	4/4	Counts	DESeq2	Discovery
GSE84896	Breast	BT474	Lapatinib	2/2	FPKM	CuffDiff	Discovery (excluded)
GSE255958	NSCLC	PC9	Osimertinib	3/3	TPM	—	Validation

Four discovery datasets spanning lung (NSCLC), colorectal, pancreatic, and melanoma DTP models were used for cross-dataset recurrence analysis. GSE84896 (breast) was included in exploratory analyses but excluded from signature generation due to technical differences in the quantification pipeline (CuffDiff FPKM with q-values). GSE255958 served as an independent external validation cohort. DTP/Ctrl indicates the number of drug-tolerant persister and parental/control samples per dataset. DE Method indicates the differential expression framework applied (limma with empirical Bayes, DESeq2 with Wald testing, or CuffDiff).

Datasets were selected against pre-specified eligibility criteria designed to maximise comparability and minimise platform-driven artefacts: (i) RNA-seq performed on Illumina platforms; (ii) an explicit, well-annotated comparison between a drug-tolerant persister (or stress-induced quiescent) population and a matched parental/control population; (iii) availability of gene-level expression data (raw counts or a normalised matrix) suitable for harmonisation; and (iv) coverage of distinct cancer lineages and DTP-inducing stressors so that any recurrent signal would reflect cross-context conservation rather than a single model. Microarray series, datasets lacking a clearly defined persister/control contrast, and series without retrievable gene-level matrices were excluded. We acknowledge that the number of qualifying datasets is modest and that this constrains the precision of any generalisability claim. The recurrence-based design partially mitigates this limitation, because a gene must satisfy the differential-expression criteria independently in every discovery dataset (4/4) to enter PanDTP-12, which makes signature membership conservative with respect to dataset-specific noise. Nevertheless, the small number of series, their derivation from established cell-line models rather than patient material, and the over-representation of certain lineages and stressors introduce potential selection bias; PanDTP-12 should therefore be regarded as a recurrently perturbed biological-state marker derived from the available public data rather than a population-representative biomarker, and broader validation across additional datasets and clinical specimens will be required to establish generalisability.

### 2.2 Data preprocessing and gene identifier harmonization

All analyses were carried out using the statistical programming language R *v*4.3.2; (R Core Team, 2023). Expression data and sample-level metadata were downloaded from GEO via GEOquery *v*2.78.0; (Davis and Meltzer, 2007). Gene-level expression data were downloaded from supplemental GEO files, where available (ideally as raw count matrices), and from GEO series matrix files otherwise. HGNC gene symbols were assigned to gene IDs using packages org. Hs. e.g.,. db (*v*3.22.0) and AnnotationDbi (*v*1.72.0). Where Ensembl gene IDs were provided, version numbers were stripped prior to mapping. Unmappable or ambiguously mappable genes were excluded. Multiple copies of gene symbols were collapsed using the average for normalized matrices and the sum for count matrices. Metadata were curated to assign each sample to one of two categories (“control” or “dtp”). In cases where count or normalized expression data were available, these were logarithmically transformed to log_2_ (x + 1).

### 2.3 Differential expression analysis

For differential expression analysis, each dataset was individually tested, comparing DTP samples against the controls, allowing for the minimization of batch effects across studies and use of recurrence analysis to detect conserved signals. The choice of statistical analysis was determined by the type of data available for each particular study. FPKM values and q-values obtained from the CuffDiff analysis were used for GSE84896. The log_2_-fold change was defined as the log_2_ ratio of persister to parental. The limma (*v*3.66.0) software was applied to the GSE153183, GSE189764, and GSE214537 datasets; it uses robust empirical Bayes moderation. A linear regression analysis was carried out using only the group variable (control vs. DTP). DESeq2 (*v*1.50.2) with median-of-ratios normalization and Wald testing was employed to analyze the raw count data of GSE246690. Multiple testing adjustments were performed using the Benjamini–Hochberg FDR correction for all datasets. Physiologically relevant DEGs were selected from those with an FDR less than 0.001 and |log_2_FC| ≥ 0.8. This strategy allows us to reliably filter false positives from our results. The lists of DEGs for further cross-dataset analysis were obtained from all the above datasets. The same significance thresholds (FDR <0.001 and |log_2_FC| ≥ 0.8), recurrence rule (4/4 across discovery datasets), module-score construction, and ROC/effect-size validation criteria were applied identically to every cancer type; no cancer-specific thresholds were used. The only context-dependent choice was the differential-expression engine, which was matched to the data type supplied by each series rather than to the cancer type: precomputed CuffDiff FPKM with q-values for GSE84896, limma with empirical-Bayes moderation for the FPKM/TPM matrices (GSE153183, GSE189764, GSE214537), and DESeq2 with median-of-ratios normalisation and Wald testing for the raw-count data (GSE246690). This deliberate harmonisation ensures that recurrence reflects shared biology across lineages rather than differing analytical settings, and the DE framework used for each dataset is recorded explicitly in Table 1.

### 2.4 Cross-dataset integration and PanDTP-12 signature derivation

DEGs identified from four discovery datasets (GSE153183, GSE189764, GSE214537, GSE246690) were combined using a recurrence-based approach to identify genes recurrently deregulated in DTPs. These four discovery datasets were GSE153183 (PC9 non-small cell lung cancer; DMSO control vs. osimertinib-induced persisters; n = 2 per group), GSE189764 (MIA PaCa-2 pancreatic cancer; control vs. Torin1/gemcitabine-induced persisters; n = 3 per group), GSE214537 (RKO colorectal cancer; standard culture vs. fasting/nutrient-restriction-induced quiescence; n = 3 per group), and GSE246690 (melanoma; proliferating cells vs. INK128-induced diapause-like persisters; n = 4 per group). Together they span four cancer lineages and four distinct DTP-inducing stressors and constitute the only series carried into the recurrence step; GSE84896 (breast, lapatinib) was used only for exploratory DEG screening and was not part of PanDTP-12 derivation, and GSE255958 (lung, osimertinib) was reserved exclusively for external validation. In this regard, the occurrence value for each gene was defined as the number of datasets that met the DEG criteria. Based on the gene occurrence table, the number of occurrences was used to rank genes. Three complementary strategies of integration were applied: (i) strict recurrency in all four datasets; (ii) robust recurrency characterized by the existence of the gene in most datasets (at least 3/4); and (iii) ranking of genes by total number of occurrences of each gene meeting the DEG criteria. The list of 12 genes representing the PanDTP-12 signature comprises genes that satisfy the DEG criteria across all four discovery datasets (recurrence 4/4), as shown in Table 2. These genes include ARSD, CLK1, DNAJB4, KLF5, MXI1, PBX1, PNP, RGS2, SAT1, SKIL, USP11, and XBP1 (Table 2).

**TABLE 2:** Log_2_ fold-change (log_2_FC) values for each of the 12 PanDTP-12 genes across the four discovery datasets.

Gene	Full name	GSE153183 (lung)	GSE214537 (CRC)	GSE189764 (Pancr.)	GSE246690 (Melan.)	Mean |FC|	Concordance
KLF5	Kruppel like factor 5	−3.49	1.40	2.75	2.16	2.45	3/4
PBX1	PBX homeobox 1	1.36	−2.06	−2.81	2.41	2.16	2/4
RGS2	Regulator of G protein signaling 2	1.06	3.46	2.37	−1.74	2.16	3/4
SAT1	Spermidine/Spermine N1-Acetyltransferase 1	0.82	3.19	2.10	2.36	2.12	4/4
DNAJB4	DnaJ HSP40 member B4	−1.24	3.44	1.21	−0.91	1.70	2/4
CLK1	CDC like kinase 1	0.80	2.78	1.48	1.12	1.55	4/4
PNP	Purine nucleoside phosphorylase	−1.09	1.22	1.25	−1.88	1.36	2/4
MXI1	MAX interactor 1	−1.93	1.04	0.96	1.33	1.31	3/4
ARSD	Arylsulfatase D	0.96	1.04	1.49	1.75	1.31	4/4
XBP1	X-box binding protein 1	0.88	1.86	1.21	−0.83	1.20	3/4
USP11	Ubiquitin specific peptidase 11	1.00	1.02	1.62	0.88	1.13	4/4
SKIL	SKI like proto-oncogene	−0.94	1.51	1.15	0.89	1.12	3/4

Positive values indicate upregulation in DTP cells; negative values indicate downregulation. Genes are ranked by mean absolute log2FC. Concordance indicates the number of datasets in which each gene shows expression in the majority direction; a subset of genes displays context-dependent directionality rather than strict uniform conservation (e.g., 4/4 = all datasets agree on direction). Note that all 12 PanDTP-12 genes satisfy the DEG selection criterion (FDR <0.001 and |log2FC| ≥ 0.8) in each of the four discovery datasets (recurrence 4/4); concordance values below 4/4 reflect context-dependent directional heterogeneity, not a failure to meet significance thresholds. The signature captures genes involved in transcriptional regulation (KLF5, MXI1, SKIL, PBX1, and XBP1), protein homeostasis (DNAJB4 and USP11), cell signaling (RGS2 and CLK1), and metabolic adaptation (SAT1, PNP, and ARSD).

**FIGURE 1:**
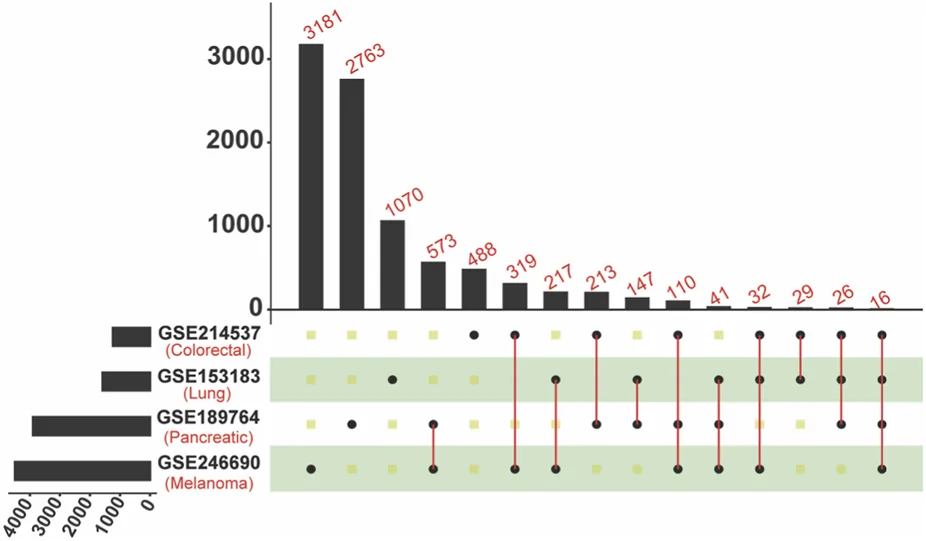
Cross-dataset recurrence of differentially expressed genes. UpSet plot summarizing the overlap of significant DEGs across discovery datasets. Vertical bars indicate the number of genes in each intersection set. The rightmost intersections represent genes meeting DEG criteria across all datasets.

The criteria for PanDTP-12 were based on recurrent differential expression across the four discovery datasets rather than strict directional uniformity. Many PanDTP-12 genes showed concordant directional behavior across models, but a subset exhibited context-dependent up- or downregulation across cancer types and stressors. PanDTP-12 should therefore be interpreted as a set of genes whose expression is consistently and substantially perturbed during DTP emergence, with the direction of change being context-dependent for a subset of members. The intersection structure between the datasets was shown in an UpSet plot generated with ComplexUpset (v1.3.3) (Figure 1). GSE84896 was part of the initial DEG identification screening process and was included in the broader set of 106 genes that were significantly differentially expressed. However, due to technical differences in expression quantification (precomputed CuffDiff FPKM values vs. the limma/DESeq2 methods used for the other datasets), it was excluded from the integration process.

### 2.5 PanDTP-12 module score computation

Module scores were generated to quantify the coordinated expression of the PanDTP-12 signature as a single quantitative value for each sample. First, expression data for PanDTP-12 genes present in each assessable dataset were obtained. Next, each gene was z-score normalized across all samples in the dataset (mean = 0, standard deviation = 1). Finally, the PanDTP-12 module score was generated for each sample as the average of the z-scores for all PanDTP-12 genes in the dataset: Score_i = (1/k) × Σ_j z (gene_j)_i; in which k is the number of PanDTP-12 genes in the dataset and z (gene_j)_i is the z-score for gene j in sample i. All datasets with fewer than five PanDTP-12 genes were excluded from further analysis. By applying z-scoring within each dataset, differences in scales between datasets can be eliminated, no information leaks between datasets will occur, and one scalar value per sample will be derived, representing coordinated expression of the signature genes.

### 2.6 Cross-dataset classification and generalization testing

Expression datasets with harmonized expression matrix data and confirmed presence of the 12 genes from the PanDTP-12 gene set were selected for the classification task: GSE153183 (n = 4, 2 controls + 2 DTPs), GSE189764 (n = 6, 3 controls + 3 DTPs), and GSE214537 (n = 6, 3 controls +3 DTPs). The total number of samples across the datasets is equal to n = 16. Individually-dataset discrimination was carried out through calculation of the receiver operating characteristic (ROC) curve, where the PanDTP-12 module scores were used as predictors (classifiers) and categorical variables (controls - 0; DTP - 1) as responses. Area under the curve was computed via the R library pROC version 1.18.5 (Robin et al., 2011) Pooled discrimination was performed by combining all 16 samples and computing the ROC-AUC. The strict separation was demonstrated by the fact that the maximum control score must be less than the minimum DTP score. Leave-One-Dataset-Out (LODO) cross-validation was used to evaluate the performance of individual ROC-AUC scores by removing one of the three datasets each time and fitting logistic regression (DTP status ∼ PanDTP-12 scores) to the other two datasets.

Because of perfect separation present in all data sets (maximum likelihood estimates tend to diverge in case of perfect separation), Firth penalized logistic regression was used with the logistf package (v1.26.1) (Firth, 1993), where the model was specified as follows: group_binary ∼ score + dataset. The approach yielded converging coefficient estimates, odds ratios, confidence intervals based on profile likelihoods, and a full likelihood ratio test. For effect size estimation, Cohen’s d was used for each data set. Confidence intervals for AUC were computed by bootstrapping (n = 2000), while permutations (n = 5000) were used to construct null AUC distributions for each data set. With only 16 samples available, the classification statistics reported here should be interpreted as indicators of biological separation rather than final estimates of predictive accuracy in heterogeneous clinical populations. To make the small-sample limitation explicit, permutation null distributions were summarized for each classifiable dataset and the 95th percentile null AUC was reported alongside the observed AUC in the supplement. To reduce the risk of information leakage, module-score calculation and classification were performed without re-estimating differential expression within the held-out sets; however, larger external cohorts and prospective validation will be required to define the true generalizability of PanDTP-12.

### 2.7 Gene ontology enrichment analysis

To determine biological processes enriched in the DTP state, functional enrichment analysis was done using a curated list of all recurrently expressed DEGs (genes meeting DEG criteria in ≥3/4 discovery data sets). For the GO-BP enrichment analysis, the package clusterProfiler (Wu et al., 2021) was used. The genes’ Entrez gene IDs were converted using org.Hs.eg.db (*v*3.22.0). The Benjamini–Hochberg method was applied to control for multiple testing (*q-value* < 0.05). The pathway enrichment analysis was performed using the ReactomePA package (v1.54.0). To validate functional consistency within individual data sets instead of only using the combined list of DEGs, the GO-BP enrichment analysis for individual DEG lists was done, and the most significantly enriched categories in each model were contrasted with each other through −log_10_ (adjusted *p-values*) clustered heatmaps. All enrichment results were visualized using enrichplot (v1.30.4) and ggplot2 (v3.5.1) (Wickham, 2016).

### 2.8 External validation (GSE255958)

Evaluation of the PanDTP-12 signature was performed using GSE255958, an independent NSCLC dataset containing parental PC9 samples and osimertinib day-9 persister samples. This dataset was not used for signature derivation and served exclusively for external validation. Expression data were processed using the same harmonization and z-score based module-score framework applied to the discovery datasets. In addition to gene-level differential expression and directional concordance assessment, PanDTP-12 module scores were computed for each relevant sample, and group differences were summarized using effect size, Wilcoxon testing, and ROC analysis. Because the number of samples per group was small, the external-validation statistics were interpreted as supportive evidence of generalizability rather than a definitive performance benchmark. This validation dataset was independent of the signature-building process. Log_2_FC values for genes in the PanDTP-12 signature were obtained from differential expression analysis, and their directional correspondence was subsequently validated against the discovery datasets.

### 2.9 Establishment of drug-tolerant persister cell models

The breast adenocarcinoma MCF-7 cells and the colorectal carcinoma HCT116 cells obtained from the Department of Biochemistry at King Abdulaziz University were cultured in DMEM supplemented with 10% FBS and 1% penicillin-streptomycin at 37 °C in a humid atmosphere with 5% CO_2_. The drug-tolerant persister (DTP) cell models for the two cancer cell types were developed by treating cells with 200 nM INK128 (Sapanisertib), a dual mTORC1/2 inhibitor. The cells were exposed to the treatment for 8 days, and the medium was replaced every 3 days to maintain exposure and assess drug efficacy. Such treatment strategies were chosen to induce the persister cell phenotype by providing prolonged sublethal therapeutic stress, mimicking drug tolerances observed in therapy. MCF-7 (breast) and HCT116 (colorectal) were selected on three grounds. First, they represent two cancer lineages that are part of the discovery set yet were not themselves the source of the recurrence-derived signature: breast was present only in the screening dataset (GSE84896), which was excluded from PanDTP-12 derivation, and colorectal entered discovery only as a fasting-induced quiescence model (GSE214537), so confirming the signature in pharmacologically induced breast and colorectal persisters provides validation that is independent of the data used to build it. Second, both are well-characterised, genomically distinct lines (HCT116 is microsatellite-unstable and KRAS-mutant; MCF-7 is hormone-receptor-positive and largely chromosomally stable), allowing the signature to be tested against contrasting genetic backgrounds. Third, both reliably enter a reversible, slow-cycling persister state under sustained mTOR inhibition, making them tractable INK128-driven DTP models that parallel the diapause-like state captured in the melanoma discovery dataset. We emphasise that these two lines were used to test whether PanDTP-12 is inducible and detectable in additional lineages, not to demonstrate mechanistic equivalence across all five cancer types; extrapolation to lung, pancreatic, and melanoma persisters rests on the cross-dataset recurrence and external validation rather than on direct wet-lab assays in those lineages, and confirmation in lineage-matched models remains future work.

### 2.10 Cell viability assay

The cytotoxicity of INK128 on MCF-7 and HCT116 cells was evaluated using the MTT [3-(4,5-dimethylthiazol-2-yl)-2,5-diphenyltetrazolium bromide] test. Cells were inoculated into 96-well plates at appropriate densities, treated with 200 nM INK128 for 72 h, and cell viability was assessed relative to untreated controls. This short-term assay was performed to confirm the sublethal nature of the selected concentration prior to the extended 8-day DTP induction protocol used for all subsequent experiments. The comprehensive process is outlined in Supplementary Methods S1.

### 2.11 Cell-cycle analysis

Both MCF-7 and HCT116 cells were plated in T25 culture flasks at a density of 1×10^5^ cells at 37 °C and 5% CO_2_ until 80% confluence. Cells were treated with INK128 200 nM for eight days; after every three days, the medium with the drug was replaced. After the treatment, cells were isolated, washed twice with 1× PBS solution, and pelleted by centrifugation. PI staining and image flow cytometry were used to detect the distribution of cell cycle stages. DTP and non-treated controls were tested on identical experimental conditions to ensure methodological reliability. Cell pellets were fixed by placing them in 70% ethanol and storing them for 30 min at −20 °C. The cells were then washed twice with PBS, followed by resuspension in 200 μL of PBS and incubation of the samples in 5 μL of propidium iodide in the dark for 30 min at room temperature. Image flow cytometry acquisition was conducted on the Amnis FlowSight imaging flow cytometer from Cytek Biosciences. Cell cycle analysis was performed using FCS Express and the MultiCycle AV program.

### 2.12 Morphological assessment

Morphological alterations in control and INK128-treated DTP cells were assessed via phase-contrast microscopy utilizing an inverted light microscope. Both MCF-7 and HCT116 cells were photographed after the eight-day treatment period. Representative photos were obtained at a uniform magnification to qualitatively assess cellular shape, intercellular spacing, and growth trends between control and DTP populations. Scale bars denote 100 μm.

### 2.13 Reverse transcription quantitative PCR (RT-qPCR)

To validate the successful establishment of drug-tolerant persister cells model and to experimentally confirm bioinformatics-predicted gene expression changes, RT-qPCR analysis was performed in the MCF-7 and HCT116 cell lines. Cells were seeded into T25 flasks at a density of 1 × 10^5^ cells per flask and treated with 200 nM INK128 as previously reported. Total RNA was extracted using the RNeasy Mini Kit (Thermo Fisher Scientific), and RNA concentration and purity were assessed using a NanoDrop spectrophotometer. cDNA was synthesized using a Thermo Fisher Scientific cDNA Synthesis Kit, and quantitative PCR was carried out using RealQ Plus 2× MasterMix Green (Ampliqon, Denmark) with gene-specific primers. Expression of the dormancy marker CDKN1B (p27) was quantified alongside twelve PanDTP-12 signature genes (PBX1, ARSD, CLK1, DNAJB4, SAT1, SKIL, KLF5, USP11, MXI1, XBP1, RGS2, and PNP) to confirm induction of the persister phenotype and validate transcriptomic alterations. Gene expression levels were normalized to GAPDH, and relative fold changes were calculated using the 2^−^ΔΔCt method (Livak and Schmittgen, 2001). Statistical significance was assessed using one-sided unpaired Student's t-tests, as the bioinformatics analysis across independent public datasets provided an *a priori* directional hypothesis of upregulation for all tested PanDTP-12 genes; importantly, the hypothesis-generating transcriptomic datasets and the hypothesis-testing wet-lab validation were conducted in different cell lines and experimental systems, mitigating circularity concerns. All experiments were conducted in triplicate using independent biological replicates, and concordance between predicted and experimentally observed expression trends was evaluated for each gene in both cell lines. Additionally, INK128 was also used as the DTP-inducing agent in one discovery dataset (GSE246690), but the wet-lab validation was performed in breast and colorectal models rather than melanoma, and INK128-specific effects limited to a single discovery dataset would not satisfy the four-of-four recurrence criterion used to define PanDTP-12.

### 2.14 Drug repurposing analysis

Pharmacological interventions targeting the DTP transcriptional regulatory network were identified through a drug-repurposing approach using two distinct strategies. Firstly, the PanDTP-12 signature genes were analyzed in the Drug–Gene Interaction Database (DGIdb v4.0; (Freshour et al., 2021)) via the REST API v2 (https://dgidb.org/api/v2/; database version 4.0 as described in Freshour et al. (2021)) to identify existing drug–gene interactions, interaction types, and gene druggability. Secondly, the recurrent DEGs set (DEGs ≥3/4 discovery datasets) was subjected to enrichR v3.2; (Kuleshov et al., 2016) to conduct gene set enrichment analysis on three pharmacogenomic data sets: DSigDB (Yoo et al., 2015), Drug_Perturbations_from_GEO (both up and down), and LINCS_L1000_Chem_Pert_Consensus_Sigs (Subramanian et al., 2017). Fisher’s exact test with Benjamini–Hochberg FDR correction (p < 0.05) was utilized to determine the significance of the enrichments. The outputs of these analyses were used to identify a focused set of actionable drug-gene interactions involving PanDTP-12 members and to contextualize the broader overlap between recurrent DTP genes and pharmacogenomic perturbation signatures. Because large numbers of nominally significant signatures can arise from overlap with generic stress-response programs, interpretation was restricted to interactions with both statistical support and mechanistic plausibility.

### 2.15 Statistical analysis

Unpaired Student’s t-tests were performed to analyze differences between DTP and control groups for qPCR, cell viability (MTT assay), and cell cycle analysis. A one-sided t-test was applied to verify the PanDTP-12 signature, as the bioinformatics analysis provided a directional hypothesis of upregulation for all tested genes, justifying a one-tailed approach. Shapiro–Wilk and Levene’s tests were conducted to assess the assumptions of normality and homoscedasticity before applying the t-test. These assumption checks were applied only to the wet-lab readouts analysed by parametric t-tests (RT-qPCR fold changes, MTT viability, and cell-cycle distributions), where the t-test depends on approximate normality and equal variance between control and DTP groups; in all comparisons the data were consistent with these assumptions (Shapiro–Wilk and Levene’s p > 0.05), so the parametric t-test was retained rather than substituted with a non-parametric alternative. The confidence intervals and AUC metrics reported elsewhere belong to the bioinformatic module-score analysis, which uses rank-based ROC estimation and bootstrap/permutation resampling and therefore does not rely on these normality and homoscedasticity assumptions; the assumption tests were consequently not used to gate the AUC or confidence-interval calculations. Given the exploratory nature of the validation and the small number of comparisons (12 genes), nominal p-values are reported without Bonferroni correction; all wet-lab procedures were performed in triplicate (independent biological replicates). The results are presented as mean ± SD. Formal power analysis was not conducted because this study aimed to generate hypotheses rather than test them. Statistical significance was set at p < 0.05.

### 2.16 Software and packages

All computational analyses were performed in R *v*4.3.2; (R Core Team, 2023). Key packages included: tidyverse (*v*2.0.0), GEOquery (*v*2.78.0), limma (*v*3.66.0), DESeq2 (*v*1.50.2), pROC (*v*1.18.5), logistf (*v*1.26.1), matrixStats (*v*1.5.0), ggplot2 (*v*3.5.1), ggpubr (*v*0.6.2), pheatmap (*v*1.0.13), ComplexUpset (*v*1.3.3), clusterProfiler (*v*4.18.4), ReactomePA (*v*1.54.0), enrichplot (v1.30.4), org.Hs.eg.db (*v*3.22.0), AnnotationDbi (*v*1.72.0), SummarizedExperiment (*v*1.40.0), enrichR (*v*3.2), and httr (*v*1.4.7). Drug–gene interactions were queried via the DGIdb *v*4.0 REST API. Complete session information is provided in Supplementary Material S1.

## 3 Results

### 3.1 Identification of PanDTP-12: a recurrently perturbed 12-gene DTP signature

**FIGURE 2:**
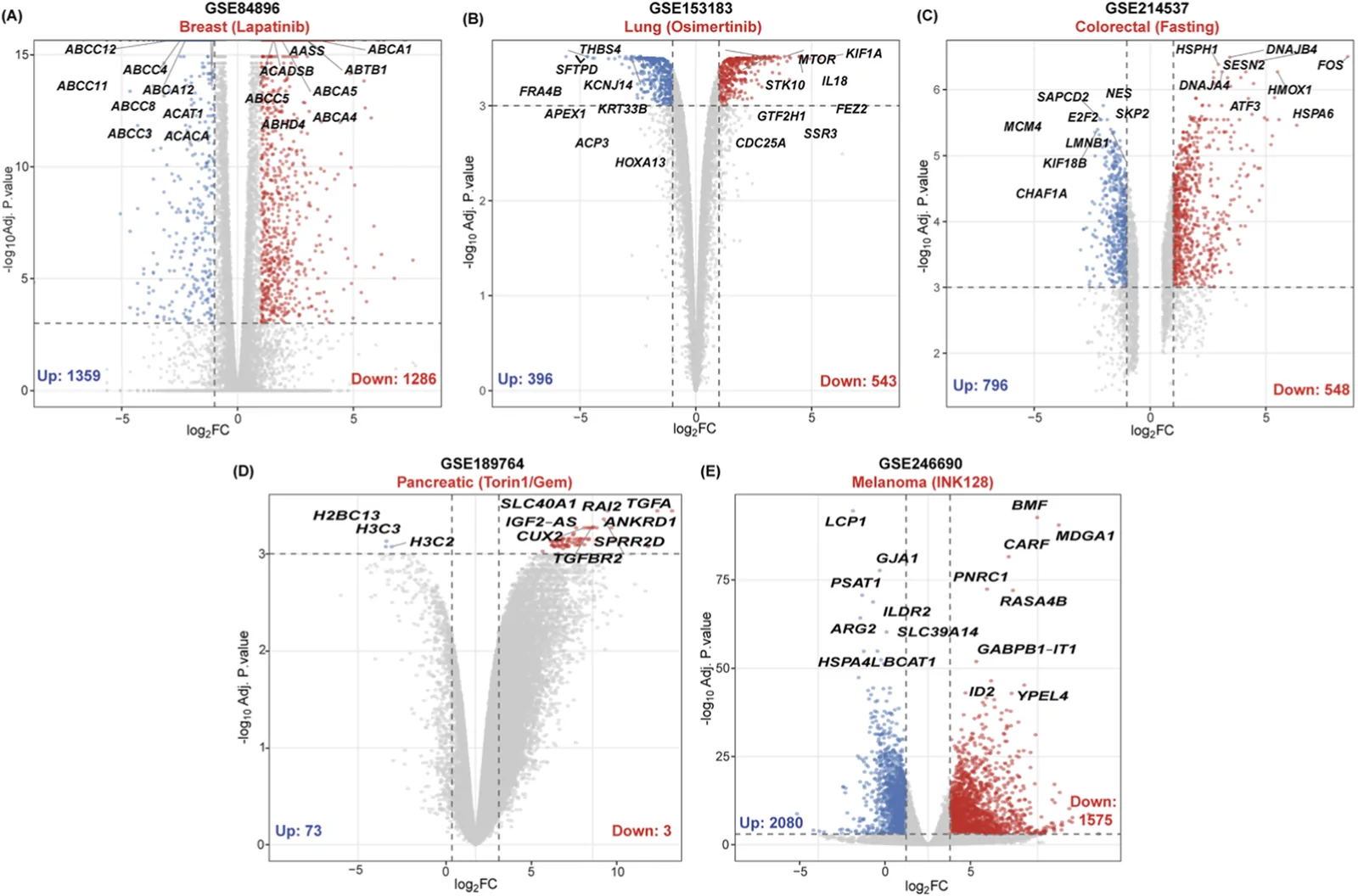
Differential expression landscape across five DTP models. Multi-panel volcano plots for each discovery dataset comparing DTP versus control samples.Red and blue dots indicate significantly upregulated and downregulated genes, respectively. Grey dots denote non-significant genes. DEGs were defined using adjusted p < 0.001 and |log2FC| ≥ 0.8. Panels show **(A)** breast (GSE84896, lapatinib), **(B)** lung (GSE153183, osimertinib), **(C)** colorectal (GSE214537, fasting), **(D)** pancreatic (GSE189764, Torin1/gemcitabine), and **(E)** melanoma (GSE246690, INK128).

Differential expression analysis was performed separately for each of the five discovery datasets, contrasting DTP samples with control samples. The research revealed multiple significantly differentially expressed genes (DEGs) across all models, indicating that DTP induction triggers extensive transcriptome remodeling irrespective of cancer type or stressor. The GSE84896 dataset (breast, lapatinib) contained 21,883 genes, of which 4,334 were significant DEGs (2,396 downregulated and 1,938 upregulated). The GSE153183 dataset (lung, osimertinib) contained 17,829 genes, resulting in 4,256 DEGs (1,995 upregulated and 2,261 downregulated). The GSE189764 dataset (pancreatic, Torin1/gemcitabine) had 19,368 genes and yielded 3,960 DEGs (1,463 upregulated and 2,497 downregulated). The GSE214537 dataset (colorectal, fasting) contained 15,304 genes, of which 1,536 were DEGs (628 upregulated and 908 downregulated). The GSE246690 dataset (melanoma, INK128) comprised 16,906 genes, yielding 2,977 differentially expressed genes (1,264 upregulated and 1,713 downregulated). Figure 2 shows volcano plots for each dataset, revealing strong bidirectional transcriptional alterations consistent with significant transcriptome reprogramming during DTP emergence.

**FIGURE 3:**
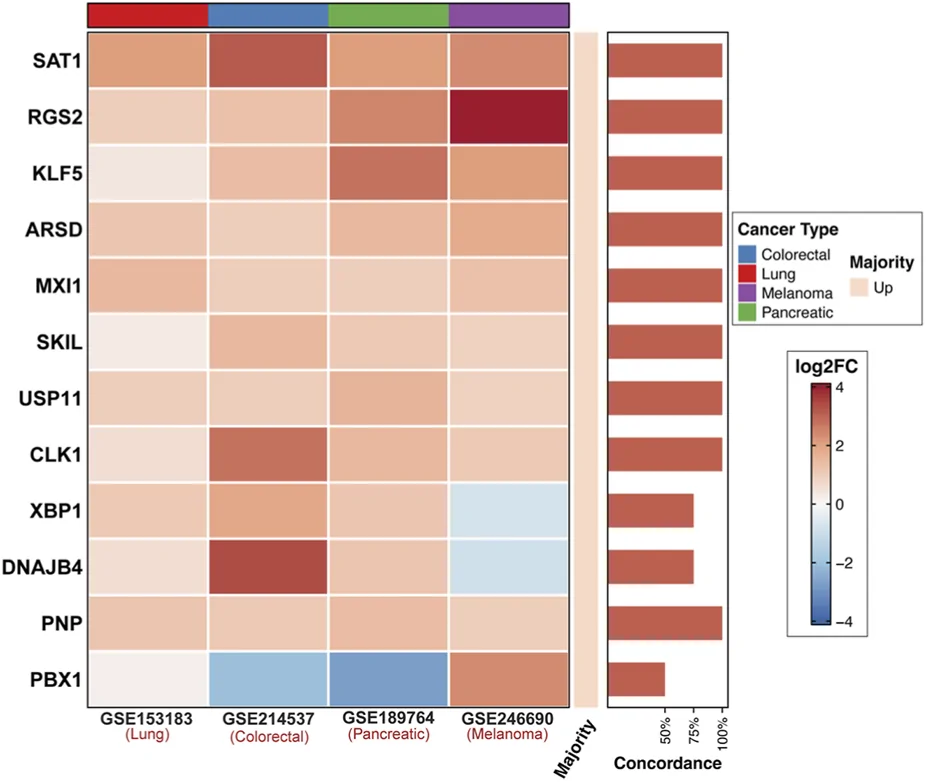
PanDTP-12 log2FC heatmap across discovery datasets. Heatmap displaying the log2 fold change (DTP vs. control) for each of the 12 PanDTP-12 signature genes across the four discovery datasets, illustrating recurrent perturbation with context-dependent directionality for a subset of members. Red indicates upregulation; blue indicates downregulation.

The recurrence analysis, limited to four discovery datasets with compatible processing workflows (GSE153183, GSE189764, GSE214537, and GSE246690), identified 12 genes that satisfied DEG criteria across all datasets, designated as the PanDTP-12 signature: ARSD, CLK1, DNAJB4, KLF5, MXI1, PBX1, PNP, RGS2, SAT1, SKIL, USP11, and XBP1 (Table 2). A log_2_FC heatmap across the discovery datasets shows that most PanDTP-12 genes are consistently oriented (Figure 3). Building on this observation, the signature was stratified into higher-concordance Core genes and more variable members (Supplementary Figure S1; Supplementary Table S1). To test whether inclusion of the fasting-induced quiescence model influenced signature derivation, the recurrence analysis was repeated using only the three pharmacologic DTP datasets; all 12 PanDTP-12 genes still met the 3/3 recurrence criterion in this drug-only analysis, indicating that the signature is not driven by GSE214537 alone (Supplementary Figure S2; Supplementary Table S2), rather than a random convergence of diverse gene lists. From Figure 1 onward, only four cancer types are displayed because the cross-dataset recurrence step, and every analysis built on it (the UpSet plot, log_2_FC heatmap, module-score classification, and per-dataset GO enrichment), is restricted to the four discovery datasets that share compatible limma/DESeq2 quantification workflows. The fifth model, GSE84896 (breast, lapatinib), was processed with a fundamentally different pipeline (precomputed CuffDiff FPKM with q-values) whose magnitude estimates are not directly comparable to count- or matrix-based moderated statistics; combining it in the recurrence count would conflate true biological recurrence with quantification-pipeline differences. GSE84896 was therefore retained only at the exploratory screening stage (contributing to the broader 106-gene set) and excluded from the integrative figures. Because PanDTP-12 was defined by a strict 4/4 recurrence rule across the four compatible datasets, the breast model’s exclusion does not alter signature membership; rather, breast biology is represented experimentally through the INK128-induced MCF-7 validation model, in which the signature was independently confirmed. We note this as a transparency point and address the resulting cross-lineage representation in the Limitations.

### 3.2 PanDTP-12 classification performance and generalization

**FIGURE 4:**
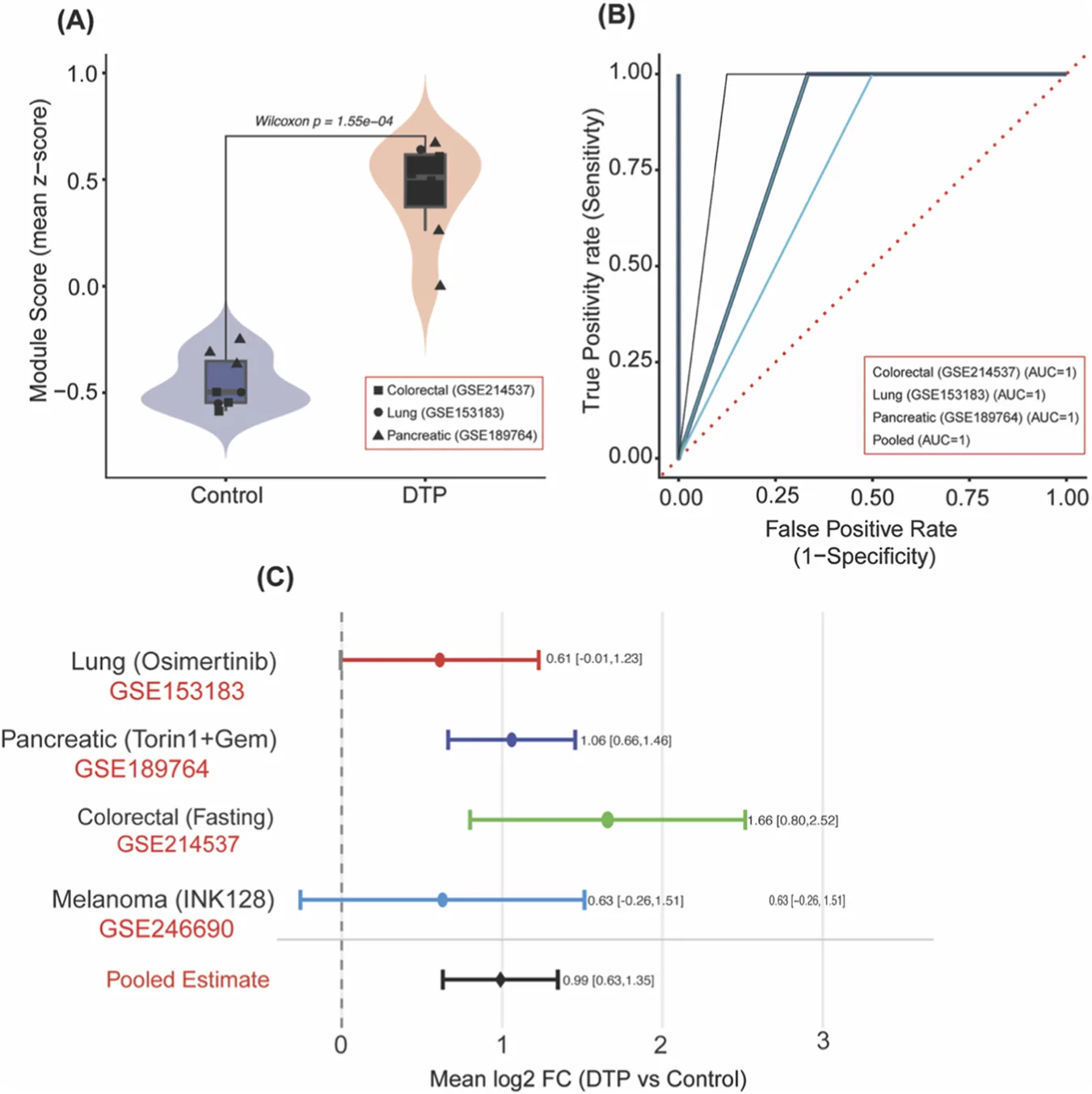
PanDTP-12 classification performance and cross-validation. **(A)** Receiver operating characteristic (ROC) curves for within-dataset and pooled classification using PanDTP-12 module scores (AUC = 1.0 for all). **(B)** Leave-one-dataset-out (LODO) cross-validation results confirming generalization (AUC = 1.0 per held-out dataset) and **(C)** Forest plot of Cohen’s d effect sizes per dataset (mean d = 1.69 ± 0.14).

A PanDTP-12 module score was computed for each sample in three evaluable datasets (GSE153183, GSE189764, GSE214537; total n = 16). In all three datasets, strict separation was observed: the maximum PanDTP-12 score among control samples was less than the minimum score among DTP samples (Figure 4). ROC analysis demonstrated perfect discrimination within each dataset (AUC = 1.0) and across datasets when pooled (AUC = 1.0). Bootstrap 95% confidence intervals for AUC were [1.00, 1.00] in all cases, reflecting the strict separation observed.

Furthermore, leave-one-dataset-out (LODO) cross-validation confirmed that PanDTP-12 generalizes across independent models. When each dataset was iteratively held out, and a logistic regression classifier was trained on the remaining datasets, AUC = 1.0 was achieved for every held-out dataset. Because complete separation yields infinite coefficient estimates in standard maximum-likelihood logistic regression, Firth penalized logistic regression was applied. This yielded a stable score coefficient of 4.61 (95% profile likelihood CI: 1.79–22.00, corresponding to an odds-ratio CI of approximately 6.0 to 3.6 × 10^9^, reflecting extreme uncertainty in the small-sample setting; *p* = 2.9 × 10^−4^). Dataset terms in the model were non-significant, indicating that discrimination is driven by the PanDTP-12 score rather than by dataset identity. The global likelihood ratio test was significant (χ^2^ = 13.1; *p* = 0.0044). Cohen’s d was large and consistent across datasets (mean d = 1.69 ± 0.14), confirming a substantial biological effect. However, permutation analyses showed that, with n = 4-6 samples per dataset, many random label permutations also produced high AUC values, reflecting the combinatorial constraints of small cohorts on possible rankings. For each dataset, the 95th percentile of the permutation null distribution was at or near 1.0 (Supplementary Figure S3; Supplementary Table S3), so formal permutation p-values are conservative and are not, by themselves, strong evidence against the null. Accordingly, the perfect observed AUC values are interpreted here primarily as indicators of a large underlying transcriptional shift, with robustness supported by convergence across effect sizes, Firth-penalized regression, sensitivity analysis, and external validation.

**FIGURE 5:**
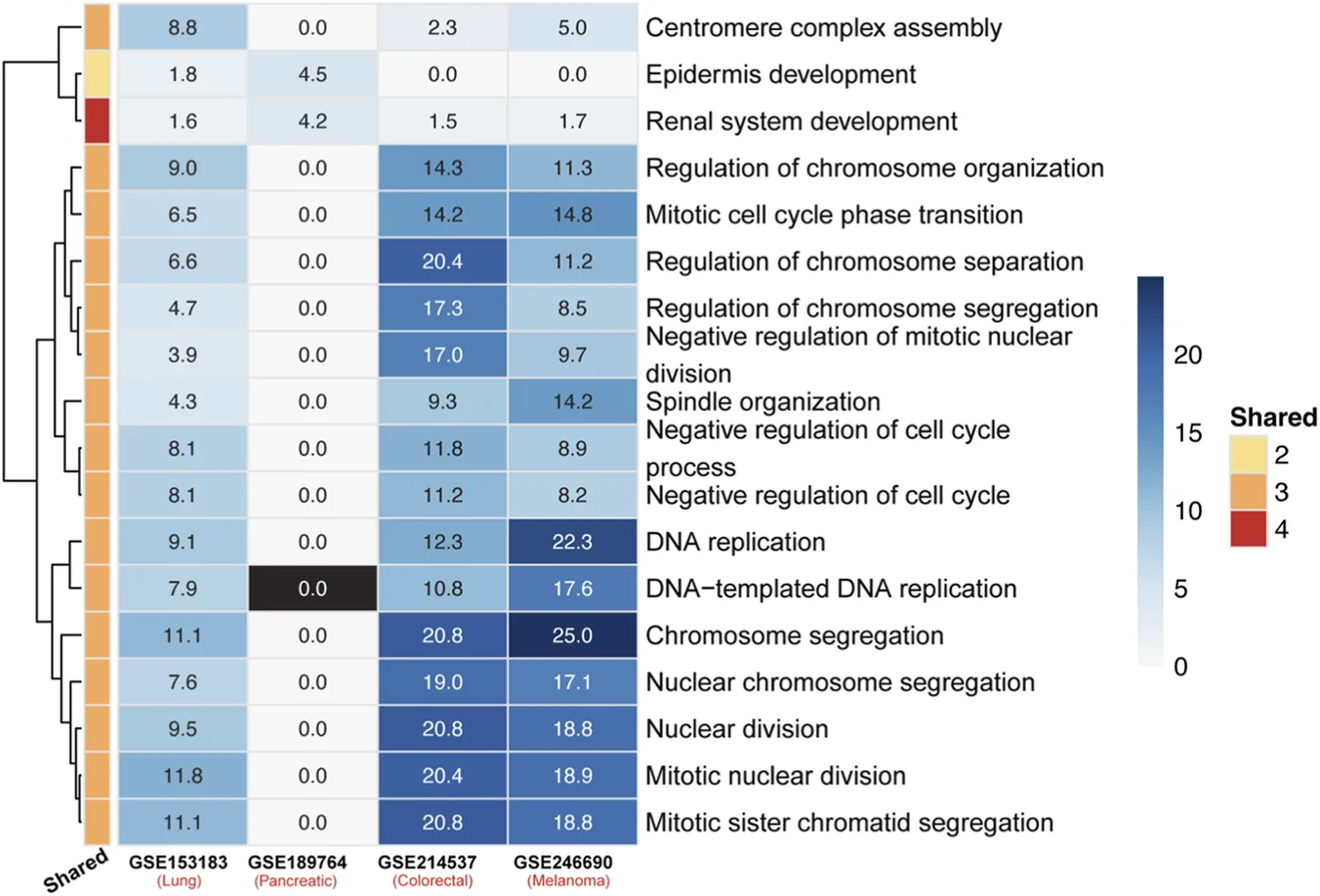
Cross-dataset consistency of GO-BP enrichment across individual DTP models. Clustered heatmap of −log_10_ (Benjamini–Hochberg adjusted p-value) for the top enriched GO-BP categories, computed independently from each discovery dataset’s DEG list (GSE153183, Lung/Osimertinib; GSE189764, Pancreatic/Torin1+Gemcitabine; GSE214537, Colorectal/Fasting; GSE246690, Melanoma/INK128). Rows represent GO-BP terms; columns represent individual datasets. Hierarchical clustering was applied to both rows and columns. Darker shading indicates stronger enrichment significance. Cell-cycle regulation, mitotic chromosome segregation, and nuclear division terms are consistently enriched across all four DTP models, confirming that proliferative shutdown is a conserved hallmark of the DTP transcriptional program independent of cancer type or therapeutic stressor.

The Gene Ontology Biological Process enrichment analysis of the larger recurrent gene set (≥3/4 datasets) showed that mitotic chromosome segregation, sister chromatid segregation, nuclear division, DNA replication, cell-cycle phase transition, spindle organization, and chromosome separation were all significantly enriched (Figure 5). These terms converge on a unifying biological theme: the mitotic/proliferative machinery that is coordinately downregulated in quiescent DTP states. An independent GO-BP enrichment analysis conducted for each dataset’s DEG list validated that cell-cycle and mitotic terms are enriched across all DTP models (Figure 5). This offers substantial evidence that DTP adaptation entails a conserved proliferative shutdown program (Rehman et al., 2021; Dhimolea et al., 2021).

### 3.3 External and experimental validation of PanDTP-12

**FIGURE 6:**
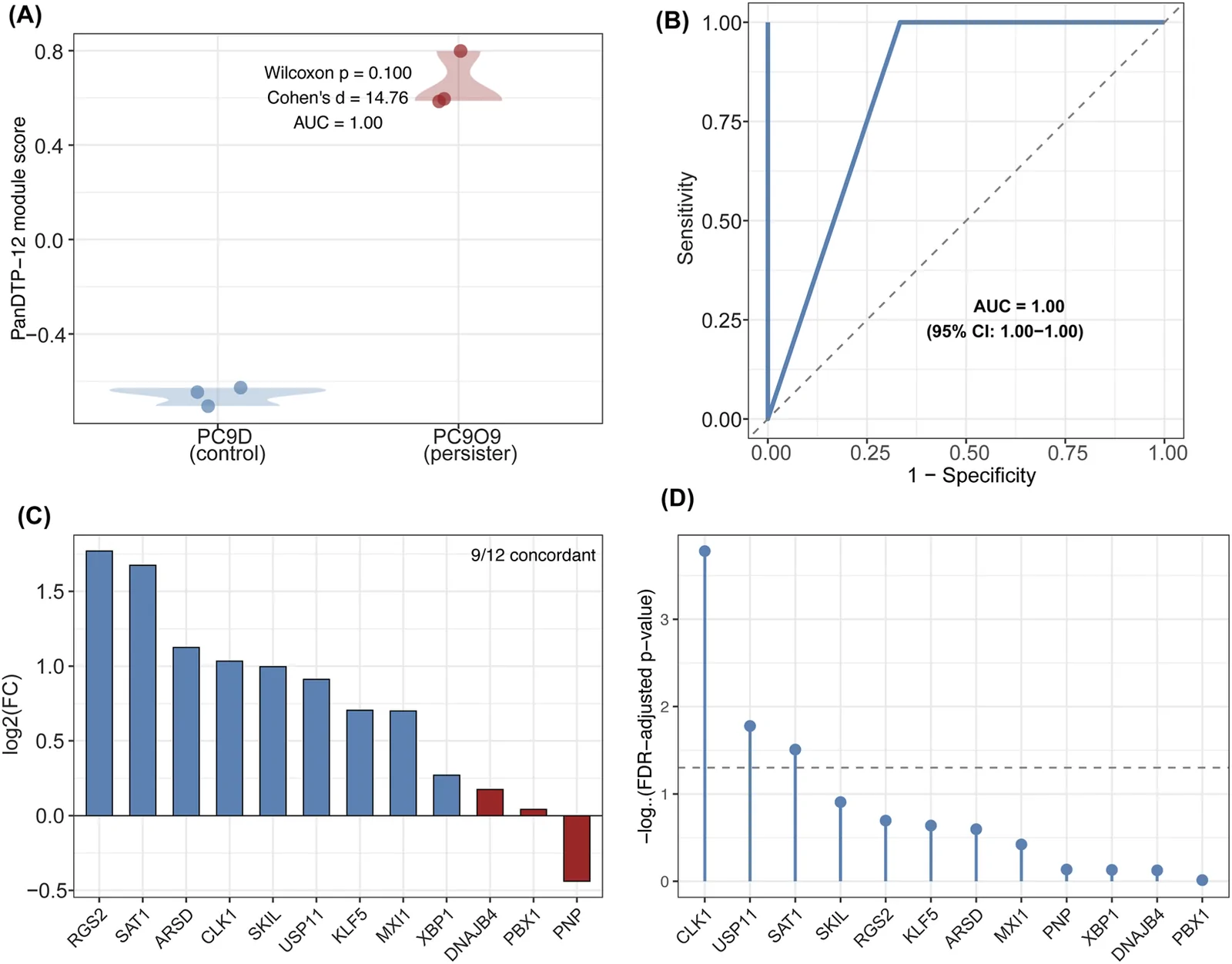
Corrected quantitative external validation of PanDTP-12 in GSE255958. **(A)** Violin and swarm plot of PanDTP-12 module scores in parental PC9D (n = 3) versus day-9 osimertinib-persister PC9O9 (n = 3) samples (Wilcoxon rank-sum p = 0.100; Cohen’s d = 14.76; AUC = 1.00). All in-panel statistical values are reported in standard notation (p = value; FDR-adjusted p where indicated), and decimal points are used throughout (e.g., p = 0.001 rather than 0:202 or 0.202); the per-gene significance values in panel **(D)** are FDR-adjusted p-values plotted as −log_10_(FDR). **(B)** ROC curve with 95% bootstrap confidence interval. **(C)** Per-gene waterfall plot showing directional concordance with the discovery consensus (9/12 concordant with the per-gene discovery-consensus direction; the three split-direction Variable genes PBX1, DNAJB4 and PNP are shown in red, PNP being the only gene moving downward consistent with its downregulation in the MCF-7 RT-qPCR) and **(D)** Lollipop plot of -log10(FDR-adjusted p-value) for each PanDTP-12 gene in the corrected differential expression analysis.

In the independent validation dataset GSE255958 (PC9 NSCLC, osimertinib day-9 persisters versus parental PC9D), all 12 PanDTP-12 genes were represented in the samples used for external validation. Gene-level analysis showed that most PanDTP-12 genes changed in the same overall direction as in the discovery datasets, with a subset (e.g., SAT1 and USP11) remaining individually significant despite the small cohort size. PanDTP-12 module scores were elevated in day-9 persister PC9O9 samples compared with parental PC9D controls, and the corrected comparison yielded complete separation and an AUC of 1.0 in this six-sample cohort (Figure 6). Given the limited sample size, these results are encouraging but should not be over-interpreted as definitive evidence of generalizability.

**FIGURE 7:**
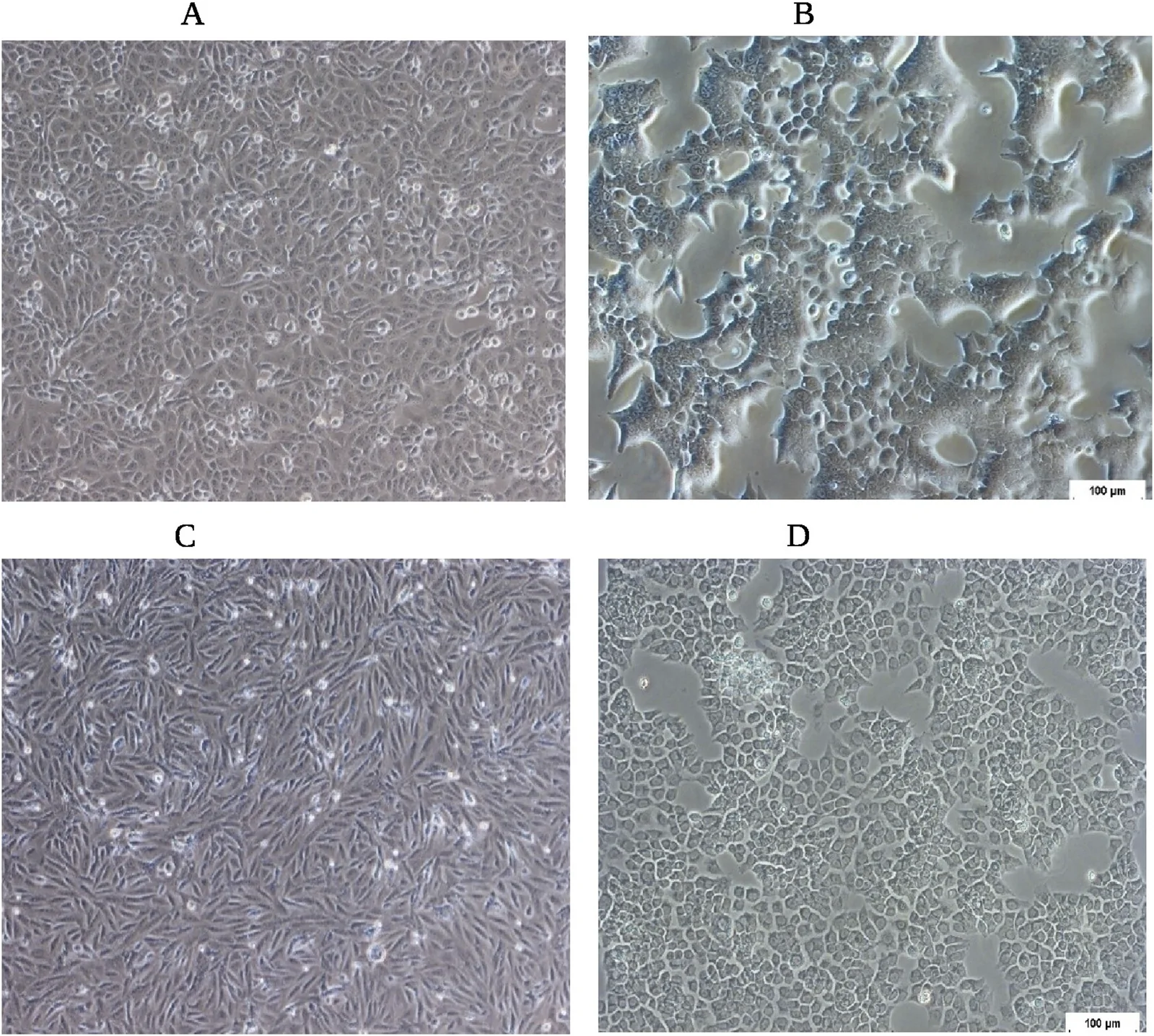
Morphological comparison between control and DTP cells. Representative phase-contrast microscopy images of **(A)** MCF-7 control, **(B)** MCF-7 DTP (INK128-treated), **(C)** HCT116 control, and **(D)** HCT116 DTP (INK128-treated) cells. DTP cells display elongated, spindle-like morphology and irregular clustering compared with epithelial-like controls. Scale bars: 100 μm.

To experimentally validate the DTP phenotype, MCF-7 and HCT116 cells were treated with 200 nM INK128 for 8 days. Cell viability, assessed by the MTT assay, remained above 80% in both models (MCF-7: 84%; HCT116: 81%), confirming that the treatment regimen induces sublethal stress that selects for surviving persister populations rather than causing bulk cell death. Morphological evaluation revealed pronounced phenotypic changes consistent with stress-induced adaptation. Untreated control cells maintained a typical epithelial-like phenotype, characterized by rounded cell shapes, tight intercellular junctions, and overall cellular uniformity in MCF-7 (Figure 7A) and HCT116 (Figure 7C) cultures. In contrast, INK128-treated DTP cells displayed a spindle-shaped, elongated morphology with irregular clustering, increased intercellular spacing, and reduced cellular uniformity in MCF-7 (Figure 7B) and HCT116 (Figure 7D) populations. These morphological changes are consistent with epithelial-to-mesenchymal-like transitions and stress-induced adaptive mechanisms that facilitate survival under sustained therapeutic pressure. Flow cytometric analysis revealed marked redistribution of cell-cycle phases in DTP cells compared with controls. In control MCF-7 cells, 53.8% were in the G1/G0 phase, 17.0% in S phase, and 15.4% in G2/M phase, indicating active proliferation. In contrast, DTP cells showed 79.5% in G1/G0 phase and a dramatic reduction to only 1.8% in S phase, with the G2/M population slightly reduced to 10.0%. Sub-G1 levels remained low in both groups. These findings demonstrate that DTP cells predominantly reside in a quiescent G1/G0-arrested state, consistent with the drug-tolerant persister phenotype and with the functional enrichment results showing downregulation of mitotic and cell-cycle progression genes (cell-cycle histograms not shown).

**FIGURE 8:**
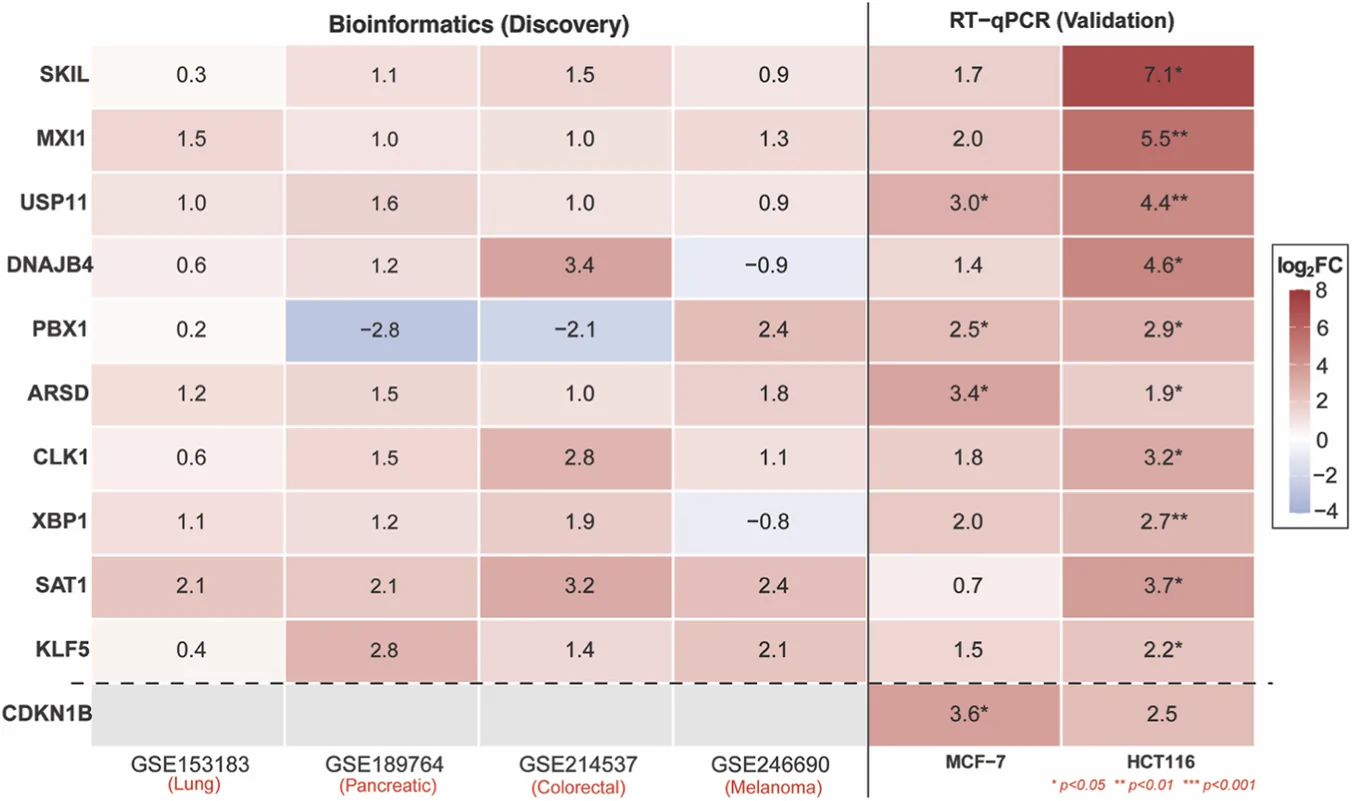
Concordance between bioinformatics predictions and qPCR validation. Heatmap displaying bioinformatics log_2_FC (four discovery datasets) alongside RT-qPCR log_2_FC for 10 PanDTP-12 genes in MCF-7 and HCT116 DTP cells. Significance annotations based on one-sided t-test: *p < 0.05, **p < 0.01, ***p < 0.001. Green shading indicates concordant upregulation; grey indicates discordance (downregulation or non-significance in one or more datasets).

**FIGURE 9:**
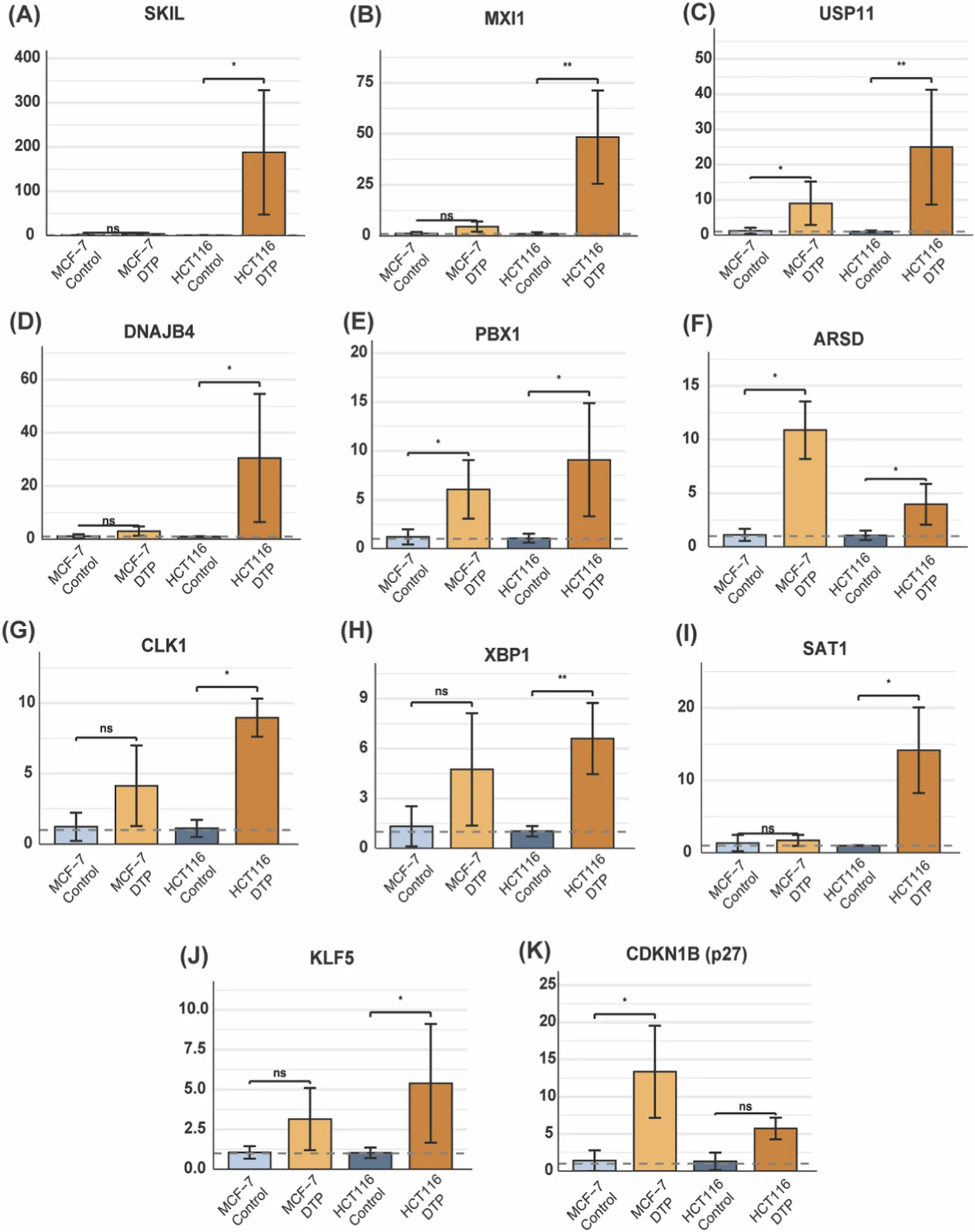
RT-qPCR validation of PanDTP-12 genes in DTP cells. Bar charts showing relative expression (2^(−ΔΔCt)^) of 10 PanDTP-12 genes in MCF-7 (blue) and HCT116 (red) DTP versus control cells. Data are presented as mean ± SD of three independent biological replicates. Significance: *p < 0.05, **p < 0.01, ***p < 0.001 (one-sided t-test). Dashed line at fold change = 1.0 indicates no change. In HCT116, 10/10 genes were significant; in MCF-7, 6/10 reached significance. RGS2 and PNP, which complete the 12-gene panel, are shown separately in Supplementary Figure S5 (with statistics in Supplementary Table S6). Panels: **(A)** SKIL, **(B)** MXI1, **(C)** USP11, **(D)** DNAJB4, **(E)** PBX1, **(F)** ARSD, **(G)** CLK1, **(H)** XBP1, **(I)** SAT1, **(J)** KLF5, and **(K)** CDKN1B (p27).

To further validate PanDTP-12 predictions, RT-qPCR was performed for all 12 PanDTP-12 genes in both cell lines (Table 3). In HCT116 DTP cells, all 12 genes were significantly upregulated (12/12; one-sided t-test, p < 0.05), with log_2_FC ranging from 1.89 (ARSD) to 7.11 (SKIL); the two newly assayed genes, RGS2 (5.28-fold, p = 0.013) and PNP (9.45-fold, p = 0.002), were also significantly upregulated, confirming their bioinformatic prediction in this lineage. In MCF-7 DTP cells, six of 12 PanDTP-12 genes reached significance (one-sided t-test, p < 0.05): ARSD (10.67-fold, p = 0.002), USP11 (7.84-fold, p = 0.011), PBX1 (5.51-fold, p = 0.018), XBP1 (4.05-fold, p = 0.050), MXI1 (3.94-fold, p = 0.037), KLF5 (2.77-fold, p = 0.037). Of note, XBP1 in MCF-7 reached only marginal significance (one-sided p = 0.050; two-sided p ≈ 0.10) and should be interpreted with caution. The dormancy marker CDKN1B (p27) was also significantly upregulated in MCF-7 DTP cells (12.3-fold, p = 0.009). When two-sided tests are applied, 3/10 MCF-7 genes remain significant (ARSD, PBX1, USP11), whereas all 10 HCT116 genes retain significance (Supplementary Table S5), consistent with the interpretation that MCF-7 shows greater inter-replicate variability rather than absence of the biological signal. The remaining four genes in MCF-7 (CLK1, DNAJB4, SKIL, and SAT1) showed directionally consistent upregulation (FC > 1 for all) but did not reach statistical significance, likely reflecting greater inter-replicate variability in MCF-7 cultures. In contrast to HCT116, RGS2 and PNP were downregulated in MCF-7 DTP cells (RGS2 0.55-fold; PNP 0.72-fold) and did not reach significance (two-sided p = 0.43 and p = 0.63, respectively); this cell-line-discordant behaviour mirrors that of PBX1 and DNAJB4 and is consistent with context-dependent directionality within the signature rather than absence of perturbation. The full RGS2 and PNP RT-qPCR results are provided in Supplementary Figure S5 and Supplementary Table S6. The data above show clear concordance between bioinformatics predictions and qPCR validation, as shown in the integrated bioinformatics–qPCR heatmap in Figure 8 and the RT-qPCR bar plots for individual genes in MCF-7 and HCT116 in Figures 9A–K. With RGS2 and PNP now assayed, all 12 PanDTP-12 members have been examined by RT-qPCR. We also note that PBX1 and DNAJB4 are downregulated in the colorectal fasting-quiescence dataset GSE214537 but upregulated in the HCT116 DTP validation model, highlighting context-dependent directionality within the signature. This apparent discrepancy is interpreted as cell-line-specific regulatory variation rather than failure of the signature, because both genes satisfy the magnitude criterion for differential perturbation in the discovery analysis and remain part of the recurrent PanDTP-12 set.

**TABLE 3:** RT-qPCR validation of 12 PanDTP-12 genes and the dormancy marker CDKN1B (p27) in INK128-induced DTP cells (200 nM, 8 days).

Gene	MCF-7 log_2_FC	MCF-7 FC	MCF-7 p-value	HCT116 log_2_FC	HCT116 FC	HCT116 p-value	MCF-7 p (2 s)	HCT116 p (2 s)	MCF-7 sig	HCT116 sig
ARSD	3.42	10.7x	0.0016	1.89	3.7x	0.0127	0.0031	0.0254	**	*
CLK1	1.81	3.5x	0.0528	3.15	8.9x	0.0018	0.1057	0.0037	ns	**
DNAJB4	1.45	2.7x	0.0653	4.62	24.6x	0.0013	0.1306	0.0026	ns	**
KLF5	1.47	2.8x	0.0369	2.21	4.6x	0.0114	0.0737	0.0227	*	*
MXI1	1.98	3.9x	0.0368	5.45	43.8x	6.20e-4	0.0737	0.0012	*	***
PBX1	2.46	5.5x	0.0185	2.86	7.2x	0.0143	0.0369	0.0285	*	*
SAT1	0.66	1.6x	0.2630	3.72	13.2x	3.80e-4	0.5260	0.0008	ns	***
SKIL	1.70	3.3x	0.0676	7.11	138.4x	7.30e-4	0.1352	0.0015	ns	***
USP11	2.97	7.8x	0.0114	4.44	21.7x	8.10e-4	0.0229	0.0016	*	***
XBP1	2.02	4.1x	0.0497	2.68	6.4x	7.90e-4	0.0993	0.0016	*	***
PNP	−0.48	0.7x	0.6855	3.24	9.4x	0.0018	0.6291	0.0035	ns (↓)	**
RGS2	−0.88	0.6x	0.7853	2.40	5.3x	0.0129	0.4293	0.0259	ns (↓)	*
CDKN1B (p27)	3.62	12.3x	0.0090	2.49	5.6x	0.0153	—	—	**	*

Log_2_FC and linear fold change (FC) were calculated using the 2^-^ᴰᴰ^c^ᵗ method with GAPDH normalization. p-values are from one-sided unpaired Student’s t-tests; two-sided p-values are also provided for transparency (n = 3 biological replicates per group). Significance: ***p < 0.001, **p < 0.01, *p < 0.05, ns = not significant. Of the 12 tested PanDTP-12 genes, 10 showed directionally consistent upregulation in both cell lines; RGS2 and PNP were upregulated in HCT116 but downregulated in MCF-7. HCT116 cells showed a stronger induction (12/12 significant), whereas MCF-7 cells showed more modest effects (6/12 significant). RGS2 and PNP were added in revision: both are significantly upregulated in HCT116 but non-significantly downregulated in MCF-7 (see Supplementary Figure S5; Supplementary Table S6).

### 3.4 Drug repurposing analysis identifies actionable targets in the DTP program

**FIGURE 10:**
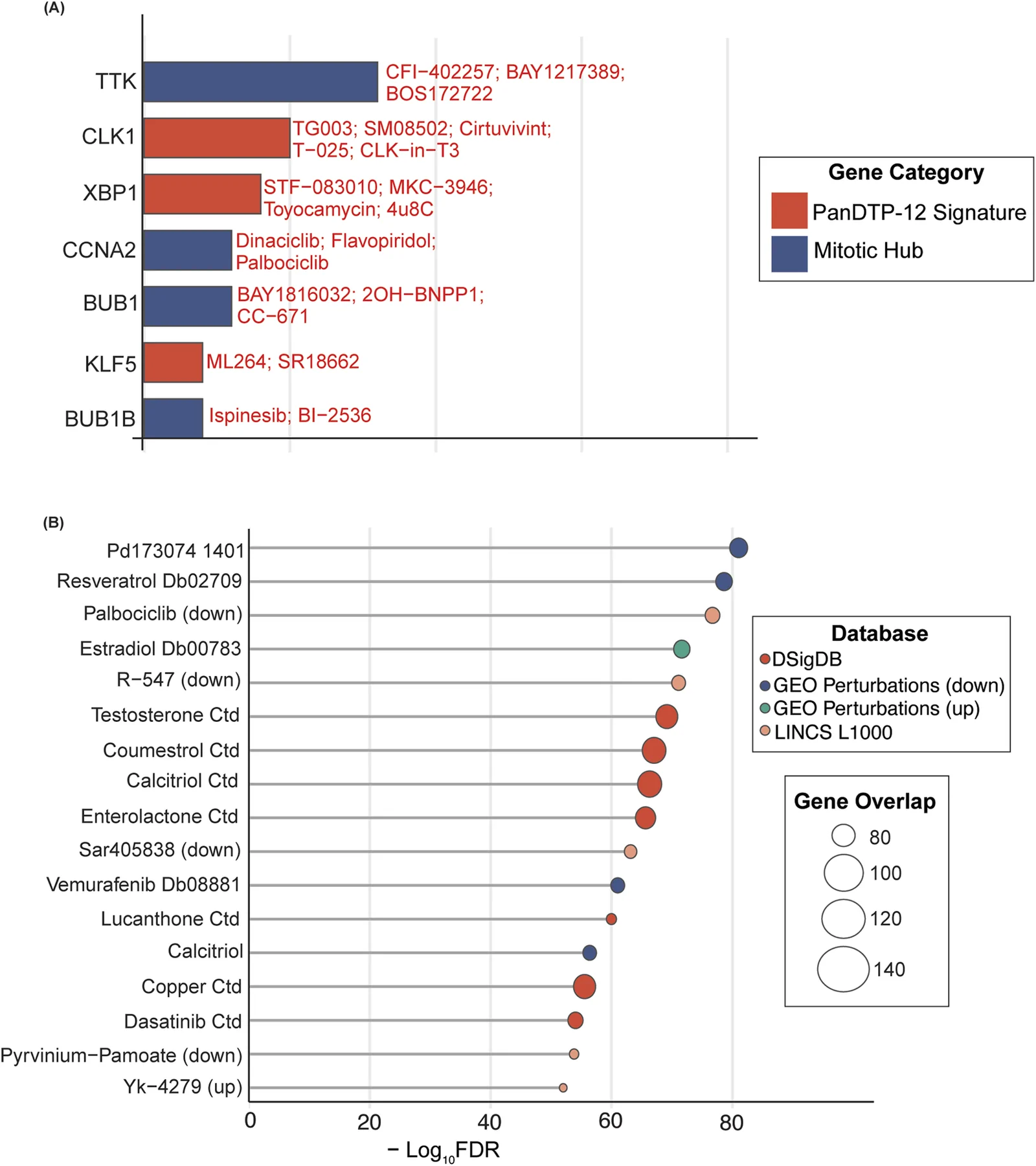
Drug repurposing analysis of DTP signature genes. **(A)** Horizontal bar chart of druggable PanDTP-12 genes with the number of known drug–gene interactions from DGIdb. Top drug names annotated per gene and **(B)** Lollipop plot of the top 20 significantly enriched drug perturbation signatures (FDR <0.05) from DSigDB, GEO Drug Perturbations, and LINCS L1000. Point size reflects gene overlap; color indicates database source.

To bridge the gap between transcriptomic discovery and therapeutic prioritization, drug-repurposing analyses were performed using both the PanDTP-12 genes and the broader set of recurrent DEGs. DGIdb queries identified three PanDTP-12 members with established drug-gene interactions: CLK1, XBP1, and KLF5, nominating these genes as the most directly actionable components of the signature (Figure 10). Gene-set enrichment analysis against pharmacogenomic perturbation resources yielded numerous nominally significant signatures, reflecting broad overlap between recurrent DTP genes and generic stress-response programs represented in these databases. Enrichment analysis of drug perturbation signatures from DSigDB, GEO Drug Perturbations, and LINCS L1000 identified a focused set of compounds whose transcriptional effects overlap with the PanDTP-12 program (Figure 10B). We therefore focused our interpretation on interactions with both statistical support and mechanistic plausibility. In this narrowed view, the DGIdb-supported targets and the strong overlap with etoposide-associated signatures were the most interpretable findings, whereas the broader list of enriched compounds should be regarded as hypothesis-generating rather than as a ranked set of DTP therapeutics.

Because several PanDTP-12 genes have been implicated in ferroptosis-related biology, exploratory enrichment analyses were conducted using curated ferroptosis gene sets. Significant enrichment of ferroptosis-associated genes was observed in the pancreatic (GSE189764, *p* = 0.0009) and melanoma (GSE246690, *p* < 0.0001) discovery datasets, whereas the signal was weaker or absent in the other models, indicating context-dependent engagement rather than a universal pan-cancer ferroptosis program (Supplementary Figure S4; Supplementary Table S4). All four PanDTP-12 members highlighted in ferroptosis-related studies, SAT1, USP11, KLF5, and XBP1, are recurrently deregulated across the discovery datasets, suggesting that modulation of ferroptosis represents a biologically plausible but still exploratory mechanistic link to the PanDTP-12 program.

## 4 Discussion

Drug-tolerant persister cells constitute a clinically significant, reversible, non-genetic pathway to therapeutic tolerance and subsequent relapse (Sharma et al., 2010; Marine et al., 2020). In various cancer contexts, DTPs consistently demonstrate cessation of proliferation, transcriptional adaptability, and stress-resilience mechanisms (Rehman et al., 2021; Dhimolea et al., 2021). A significant deficiency in the area has been the lack of compact gene signatures conserved across various cancer types and therapy settings that demonstrate resilience under stringent cross-dataset analyses. In this study, we filled this gap by systematically combining DTP transcriptomes from multiple datasets and identifying a strict 12-gene signature (PanDTP-12) that clearly characterizes DTP-like states and is consistent across models. The strength of our integrative approach lies in its conservative design: differential expression was performed independently within each dataset, eliminating cross-study batch effects, and recurrence was assessed across datasets to identify signals that are genuinely conserved rather than dataset-specific. The PanDTP-12 genes were derived from the most rigorous recurrence tier (4/4 discovery datasets), guaranteeing that each gene is strongly linked to the DTP state across various cancer types, such as lung, pancreatic, colorectal, and melanoma, and a wide range of therapeutic stressors, including osimertinib, Torin1/gemcitabine, nutrient restriction, and INK128. The directionality of PanDTP-12 genes was concordant in many but not all dataset–stressor combinations, supporting a recurrently perturbed rather than strictly directionally uniform program. This cross-cancer recurrence is biologically meaningful given the technical and biological diversity of the included models.

Recurrence of differential expression does not imply strict directional uniformity across all models. Several PanDTP-12 genes, including PBX1, DNAJB4, PNP, and KLF5, exhibit context-dependent changes in direction across datasets, likely reflecting lineage-specific regulatory programs or stressor-specific adaptation rather than a failure of the signature itself. In support of this interpretation, concordance-stratified analysis separated PanDTP-12 into a higher-concordance Core subset and a more variable subset, and the Core genes alone retained complete discrimination with equal or larger effect sizes than the full signature (Supplementary Figure S1). These findings suggest that PanDTP-12 captures both robust pan-cancer DTP nodes and a smaller set of context-responsive modifiers.

With pooled and leave-one-dataset-out AUC values of 1.0, the PanDTP-12 module score clearly captures a strong transcriptional separation between DTP and control states in the available discovery data. However, in cohorts of only four to six samples per dataset, perfect separation is not synonymous with a clinically robust classifier, because the space of possible score rankings is highly discrete, and even the null permutation distributions can yield very high AUC values. The observed AUC values, therefore, reflect the magnitude of the underlying biological signal rather than a definitive estimate of predictive accuracy in heterogeneous clinical populations. Confidence in the relevance of the signature, therefore, rests on convergence across recurrence analysis, effect sizes, Firth-penalized regression, drug-only sensitivity analysis, external validation, and wet-lab confirmation, rather than on AUC values alone. GSE214537 represents a fasting-induced quiescent state rather than a pharmacologically induced DTP model, and this distinction should be made explicit. Its inclusion was intended to capture biological overlap between DTP and reversible stress-induced quiescence, particularly G1/G0 arrest and proliferative shutdown. Importantly, repeating the recurrence analysis without GSE214537 showed that all 12 PanDTP-12 genes remained recurrent across the remaining pharmacologic models (Supplementary Figure S2; Supplementary Table S2), indicating that the signature is not an artifact of the quiescence dataset and may instead capture a broader adaptive-survival program shared across related persister-like states.

### 4.1 Limitations

At the outset we emphasise that, given the small per-group sample sizes and the complete within- and cross-dataset separation, PanDTP-12 is best regarded at present as a biological-state marker of the DTP/quiescent phenotype rather than a validated clinical diagnostic or prognostic classifier; the reported AUC values index the magnitude of the underlying transcriptional shift, not deployable predictive accuracy in heterogeneous patient populations. Several limitations warrant consideration. First, the total sample size across the three classifiable discovery datasets is modest (n = 16), and the perfect AUC values observed in this setting should not be interpreted as stable estimates of classifier performance in biologically heterogeneous populations. In particular, with n = 2 per group in GSE153183, perfect separation is mathematically near-inevitable for any moderately discriminatory score, and the wide Firth-regression confidence interval reflects this instability. The convergence of recurrence analysis, leave-one-dataset-out behavior, effect-size estimates, external validation, and wet-lab confirmation supports the conclusion that PanDTP-12 captures a real signal. Still, these lines of evidence cannot substitute for larger independent validation cohorts. Second, RGS2 and PNP have now been validated by RT-qPCR in both cell lines (Supplementary Figure S5; Supplementary Table S6). Both were significantly upregulated in HCT116 DTP cells (RGS2 5.28-fold, p = 0.013; PNP 9.45-fold, p = 0.002), confirming their bioinformatic prediction, but were non-significantly downregulated in MCF-7 (RGS2 0.55-fold; PNP 0.72-fold). This cell-line-discordant directionality—also seen for PBX1 and DNAJB4—indicates that RGS2 and PNP behave in a context-dependent manner across lineages; their inclusion in PanDTP-12 rests on recurrent discovery-stage differential expression, and their lineage-dependent qPCR behaviour should be considered when interpreting the signature. Third, the wet-lab validation relied on a single DTP-inducing agent, INK128, in two cell lines. Although this partially tests cross-lineage reproducibility, it does not replicate the full therapeutic diversity represented in the discovery analysis. Fourth, one of the discovery datasets (GSE214537) represents fasting-induced quiescence rather than a classical drug-tolerance model; however, sensitivity analysis demonstrated that the signature remains intact when this dataset is excluded, indicating that the principal findings do not depend on that model alone. More broadly, the discovery datasets are biologically and technically heterogeneous (different cell lines, stressors, quantification pipelines, and the inclusion of one fasting-induced quiescence model), and although the recurrence rule and drug-only sensitivity analysis guard against any single dataset driving the result, we therefore performed this unsupervised comparison directly. Principal component analysis of the four harmonised discovery datasets (quantile normalisation across all 54 samples to remove the log-versus-linear scale differences between series, followed by log_2_ transformation and selection of the 1,000 most variable genes) showed that the datasets separate primarily by cancer lineage along PC1–PC2 (44.6% of variance), as expected, and that GSE214537 is not an outlier: its samples show positive global rank correlation with all three pharmacologically induced models (Spearman r = 0.56–0.68), highest with the pancreatic Torin1/gemcitabine model (GSE189764, r = 0.68), and in correlation-based hierarchical clustering GSE214537 groups with the pharmacological models rather than forming a separate branch, whereas the lung osimertinib dataset (GSE153183) is the most distinct series. GSE214537 does carry some unique variance on PC3 (12.8%), consistent with fasting being a mechanistically distinct stressor; together these results support using GSE214537 as a complementary stress-induced quiescence reference that shares the persister program while testing stressor-independence (Supplementary Figure S6). Fifth, the external validation dataset is also small, and therefore supports generalizability only provisionally. Finally, the study’s ferroptosis and drug-repurposing components are hypothesis-generating and require direct functional and pharmacologic testing before translational claims can be made. Future work incorporating functional validation, patient-derived organoids, larger multi-institutional cohorts, and prospective clinical data will be essential for translating PanDTP-12 from a computational biomarker to clinical application.

The GO enrichment study of the larger set of recurrent genes showed that they were mostly involved in sister chromatid segregation, nuclear division, DNA replication, and spindle organization during mitosis (Wu et al., 2021). Additionally, no single dataset drove this enrichment. Both independent per-dataset enrichment and cross-dataset consistency analysis indicated that terms related to the cell cycle and mitosis are more common in all DTP models. This functional convergence aligns with the standard DTP trait of halting cell growth and accumulating in G1 (Rehman et al., 2021; Liau et al., 2017). The coordinated downregulation of mitotic machinery genes in DTP cells indicates that they are actively withdrawing from cell-cycle progression, a feature of the quiescent persister state. This provides a mechanistic link between the changes in gene expression and the DTP trait.

Our experimental validation offered independent confirmation for these computational results. INK128-treated MCF-7 and HCT116 cells displayed characteristic features of DTP biology: a morphological shift from an epithelial-like to a spindle-shaped phenotype, indicative of stress-induced mesenchymal adaptation; a marked accumulation in the G1/G0 phase (79.5% compared to 53.8% in controls); a near-total reduction of S-phase cells (1.8% versus 17.0%); and a significant upregulation of the canonical dormancy marker CDKN1B (p27). The RT-qPCR validation of all 12 PanDTP-12 genes directly confirmed the bioinformatics-derived signature. In HCT116 cells, all 12 examined genes were significantly elevated (12/12; one-sided t-test, p < 0.05), with fold increases ranging from 1.89 (ARSD) to 7.11 (SKIL). In MCF-7 cells, six of twelve genes were statistically significant, with ARSD (10.67-fold), USP11 (7.84-fold), and PBX1 (5.51-fold) showing the most pronounced effects. The elevated validation rate in HCT116 presumably indicates reduced inter-replicate variability rather than biological inconsistency. Ten of the twelve genes exhibited directionally consistent upregulation in both cell lines; the exceptions, RGS2 and PNP, were significantly upregulated in HCT116 but downregulated in MCF-7, a cell-line-context-dependent pattern paralleling that of PBX1 and DNAJB4. The four non-significant MCF-7 genes (CLK1, DNAJB4, SKIL, and SAT1) exhibited consistently directed upregulation, indicating that fundamental biology is preserved but that statistical significance is limited by inter-replicate variability in MCF-7. Beyond inter-replicate variance, the behaviour of these four genes is biologically coherent with their regulation and with the MCF-7 background, so the result should not be read solely as technical noise. CLK1 is a cell-cycle- and stress-responsive splicing kinase whose activity oscillates with proliferative state; in slow-cycling MCF-7 persisters its transcript induction may be buffered post-transcriptionally and by autoregulatory splicing of its own pre-mRNA, dampening the fold change measured at the mRNA level. SKIL (SnoN), a negative-feedback regulator of TGF-β/SMAD signalling, is itself a TGF-β target subject to rapid SMAD-driven turnover; in luminal, epithelial MCF-7 cells with comparatively low baseline TGF-β/EMT tone, its net induction is expected to be more muted than in mesenchymal-prone HCT116. DNAJB4, an HSP40 co-chaperone, is induced as part of a proteostatic stress response whose amplitude scales with the degree of mTOR-inhibition-driven proteotoxic and ER stress; the markedly stronger XBP1/UPR activation seen in HCT116 is consistent with a larger DNAJB4 response in that line. SAT1, a p53-responsive polyamine-catabolism and ferroptosis-linked gene, showed the smallest MCF-7 effect (1.6-fold), in keeping with its context-dependent ferroptosis engagement, which in our discovery analysis was strongest in the pancreatic and melanoma models rather than uniform across lineages. Taken together, all four genes moved in the predicted direction in both lines, and their attenuated MCF-7 significance reflects a combination of *bona fide* lineage-specific regulatory tone and limited replicate power rather than absence of the signal; this interpretation is reinforced by their robust, significant induction in HCT116.

Our drug repurposing analysis links transcriptome findings to pharmacological intervention, an angle that has received limited attention in the DTP literature (Corsello et al., 2020). Querying the DGIdb identified three druggable genes in the PanDTP-12 gene set: CLK1 (5 known drug interactions), XBP1 (4 known drug interactions), and KLF5 (2 known drug interactions). These drug-gene interactions suggest that targets upregulated in drug-tolerant persisters (e.g., CLK1, XBP1) may be inhibited to eliminate quiescent cells. Numerous identified drugs are currently in clinical use or in advanced trials: etoposide and doxorubicin are recognized chemotherapeutic agents. CLK1 inhibitors (TG003, SM08502) constitute an emerging class of splicing modulators exhibiting proven anticancer efficacy (Tam et al., 2020). XBP1 stands out as a candidate target driven, given its role as a principal effector of the unfolded protein response (UPR), and its inhibitors (STF-083010, toyocamycin) have demonstrated preclinical efficacy in models of multiple myeloma and breast cancer (Mimura et al., 2012). The clinical maturity of agents against these three nodes differs markedly, and this shapes their near-term translational value. For CLK1, no selective CLK1 inhibitor has yet reached routine clinical use; the dual CLK/DYRK inhibitor cirtuvivint (SM08502) and related splicing-kinase inhibitors have entered early-phase oncology trials, while tool compounds such as TG003 remain preclinical. CLK inhibition broadly perturbs spliceosome function, so the principal translational challenges are a narrow therapeutic window, on-target effects on normal splicing, and the likelihood of adaptive resistance through compensatory CLK/SRPK paralogues. For XBP1, the molecule itself is not directly druggable; clinical efforts target its upstream activator IRE1α (RNase inhibitors such as MKC-8866/ORIN1001, which has been evaluated in early-phase breast cancer trials), with STF-083010 and toyocamycin remaining preclinical UPR tool compounds. Because the UPR is cytoprotective but also context-dependent, anticipated liabilities include incomplete pathway blockade, switching between adaptive and terminal UPR outputs, and resistance via parallel ATF6/PERK arms. For KLF5, no clinical-stage direct inhibitor exists; reported strategies are indirect (for example, ML264 and metformin-associated KLF5 suppression) and remain experimental, and KLF5’s dual oncogenic/tumour-suppressive behaviour across lineages makes context-specific targeting a substantial obstacle. A unifying caveat is that, because persister cells survive by reversible, redundant stress-adaptive programmes, single-node inhibition of any one of these genes is likely to provoke compensatory rewiring; their main translational promise therefore lies in rational combination with the primary targeted therapy or with cell-cycle-directed agents to limit persister outgrowth, a hypothesis that will require direct functional and pharmacological testing. The pharmacogenomic enrichment analysis should be interpreted cautiously. The very large number of nominally significant perturbation signatures most likely reflects extensive overlap between recurrent DTP genes and broadly represented stress-response or proliferation-linked signatures in public drug databases, rather than a specific catalog of anti-persister compounds. The most credible translational findings, therefore, come from the focused drug-gene interactions involving CLK1, XBP1, and KLF5, together with compounds whose overlap has an immediately coherent mechanistic rationale, such as etoposide in the setting of marked cell-cycle suppression. Gene-set enrichment analysis against DSigDB, GEO drug perturbations, and LINCS L1000 returned numerous nominally significant signatures, among which etoposide showed the most coherent overlap (36/48 genes, FDR <0.001) with the recurrent DTP gene set, consistent with the strong cell-cycle suppression observed in DTP cells. The remainder of the enrichment landscape is best treated as hypothesis-generating rather than as a ranked therapeutic shortlist.

Previous studies have shown that multiple signaling pathways, particularly those involved in stress adaptation and survival, are enriched in drug-tolerant persister cells. Genome-wide CRISPR screens in EGFR-mutant lung cancer identified the YAP/TEAD axis as a key mediator of persister survival (Pfeifer et al., 2024), and a FAK–YAP signaling module was shown to promote residual disease in NSCLC models (Haderk et al., 2024). In this context, our finding that ARSD is overexpressed in DTP cells is notable, as ARSD has been reported to activate Hippo/YAP signaling in breast cancer (Lin et al., 2021), suggesting that ARSD may reinforce persister phenotypes by modulating the stress-responsive YAP pathway. Regarding the PanDTP-12 gene set specifically, accumulating evidence indicates that drug-tolerant persister cells evade therapy not through stable genetic resistance but via adaptive stress-response programs, among which suppression of ferroptotic cell death has emerged as a critical survival mechanism (Ou et al., 2016). USP11 represents a more direct ferroptosis suppressor, as it inhibits ferroptotic cell death through deubiquitination-dependent stabilization of antioxidant and epigenetic regulators, thereby promoting resistance to chemotherapy and targeted agents (Duan et al., 2023). In addition, KLF5 has been reported to function as a negative regulator of ferroptosis by limiting oxidative stress and lipid peroxidation, thereby promoting cancer cell survival and therapeutic resistance, indicating a context-dependent role for KLF5 in ferroptosis control (Jiang et al., 2024). Notably, XBP1 has been shown to suppress ferroptosis under hypoxic conditions by activating the XBP1s–MYDGF axis, which stabilizes LCN2 via UBQLN1-dependent mechanisms, thereby promoting cancer cell survival in stressful microenvironmental conditions (Zhou et al., 2026). The ferroptosis-related findings should be regarded as hypothesis-generating rather than established. Several PanDTP-12 members, including SAT1, USP11, KLF5, and XBP1, have documented links to ferroptosis-related pathways in cancer contexts, and exploratory analyses in the present work identified enrichment of ferroptosis-associated genes in a subset of discovery datasets (Supplementary Figure S4; Supplementary Table S4). However, the signal was not uniform across all models, suggesting that ferroptosis may be context-dependent rather than a universal hallmark of all DTP states. Direct functional testing will be required to determine whether ferroptosis vulnerability is a tractable liability of PanDTP-12-high persister cells. Altogether, these observations are consistent with a model in which ferroptosis suppression may contribute to DTP survival via SAT1, USP11, KLF5, and XBP1, but remain hypothesis-generating; direct functional testing in DTP models will be required before ferroptosis can be considered a recurrent feature of the persister state.

Several PanDTP-12 members have documented clinical correlations with drug response and resistance in the lineages studied here, which strengthens the translational rationale for the signature. In lung cancer, XBP1/IRE1α UPR activation has been linked to tolerance and relapse under EGFR-targeted therapy, the same osimertinib context represented by our lung discovery and validation datasets, and IRE1α blockade has been proposed to limit residual disease. KLF5 is an established driver of proliferation and treatment resistance in breast and colorectal cancer and has been associated with poorer response to chemotherapy, consistent with its appearance as a recurrent, druggable node in our analysis. SAT1, a p53-inducible polyamine-catabolism gene, modulates sensitivity to oxidative and ferroptosis-inducing agents and has been tied to chemotherapy response in colorectal and other models, while USP11 stabilises DNA-repair and antioxidant effectors and its overexpression has been correlated with resistance to PARP inhibitors and platinum agents in breast and other cancers. CLK1-dependent splicing has been implicated in resistance to targeted and cytotoxic therapy, and CLK inhibitors are being explored precisely as resensitising agents. These correlations, together with the DGIdb-supported interactions for CLK1, XBP1, and KLF5 and the coherent overlap with etoposide signatures, position PanDTP-12 not only as a biological-state marker but as a source of testable drug-repurposing and resensitisation hypotheses; however, because most evidence derives from bulk tumour or non-persister contexts, these links should be regarded as supportive rather than as established persister-specific drug-response biomarkers until validated in DTP-enriched material.

In conclusion, this study defines PanDTP-12 as a stringent, recurrently perturbed 12-gene program marking drug-tolerant persister states across multiple cancer models, with directionality that is largely concordant for a Core subset and context-dependent for a Variable subset. The signature offers a compact biomarker hypothesis, focused druggable nodes (CLK1, XBP1, KLF5), and a framework for future validation in larger cohorts and direct functional studies.

## Data Availability

The datasets analyzed in this study are publicly available from the NCBI Gene Expression Omnibus (GEO) database (https://www.ncbi.nlm.nih.gov/geo/) under the following accession numbers: GSE84896 (breast, lapatinib), GSE153183 (lung, osimertinib), GSE189764 (pancreatic, Torin1/gemcitabine), GSE214537 (colorectal, nutrient restriction), GSE246690 (melanoma, INK128), and GSE255958 (lung, osimertinib; external validation). Drug–gene interaction data were queried from the Drug–Gene Interaction Database (DGIdb v4.0; https://dgidb.org/). Drug perturbation enrichment was performed using enrichR against DSigDB, DrugPerturbationsfromGEO, and LINCSL1000ChemPertConsensusSigs databases.

## References

[B1] Barrett T. Wilhite S. E. Ledoux P. Evangelista C. Kim I. F. Tomashevsky M. (2013). NCBI GEO: archive for functional genomics data sets—update. Nucleic Acids Res. 41 (D1), D991–D995. 10.1093/nar/gks1193 23193258 PMC3531084

[B2] Bell C. C. Gilan O. (2020). Principles and mechanisms of non-genetic resistance in cancer. Br. J. Cancer 122 (4), 465–472. 10.1038/s41416-019-0648-6 31831859 PMC7028722

[B3] Corsello S. M. Nagari R. T. Spangler R. D. Rossen J. Kocak M. Bryan J. G. (2020). Discovering the anticancer potential of non-oncology drugs by systematic viability profiling. Nat. Cancer 1 (2), 235–248. 10.1038/s43018-019-0018-6 32613204 PMC7328899

[B4] Davis S. Meltzer P. S. (2007). GEOquery: a bridge between the Gene expression omnibus (GEO) and BioConductor. Bioinformatics 23 (14), 1846–1847. 10.1093/bioinformatics/btm254 17496320

[B5] Dhanyamraju P. K. Schell T. D. Amin S. Robertson G. P. (2022). Drug-tolerant persister cells in cancer therapy resistance. Cancer Res. 82 (14), 2503–2514. 10.1158/0008-5472.CAN-21-3844 35584245 PMC9296591

[B6] Dhimolea E. Simoes R. de M. Kansara D. Al’Khafaji A. Bouyssou J. Weng X. (2021). An embryonic diapause-like adaptation with suppressed myc activity enables tumor treatment persistence. Cancer Cell. 39 (2), 240–256.e11. 10.1016/j.ccell.2020.12.002 33417832 PMC8670073

[B7] Duan J. Huang D. Liu C. Lv Y. Zhang L. Chang F. (2023). USP11-mediated LSH deubiquitination inhibits ferroptosis in colorectal cancer through epigenetic activation of CYP24A1. Cell. Death Dis. 14 (7), 402. 10.1038/s41419-023-05915-9 37414755 PMC10326026

[B8] Firth D. (1993). Bias reduction of maximum likelihood estimates. Biometrika 80 (1), 27–38. 10.1093/biomet/80.1.27

[B9] Freshour S. L. Kiwala S. Cotto K. C. Coffman A. C. McMichael J. F. Song J. J. (2021). Integration of the drug–gene interaction database (DGIdb 4.0) with open crowdsource efforts. Nucleic Acids Res. 49 (D1), D1144–D1151. 10.1093/nar/gkaa1084 33237278 PMC7778926

[B10] Haderk F. Chou Y. T. Cech L. Fernández-Méndez C. Yu J. Olivas V. (2024). Focal adhesion kinase-YAP signaling axis drives drug-tolerant persister cells and residual disease in lung cancer. Nat. Commun. 15 (1), 3741. 10.1038/s41467-024-47423-0 38702301 PMC11068778

[B11] Jiang H. Zeng Y. Jiang X. Xu X. Zhao L. Yuan X. (2024). Ketogenesis attenuated KLF5 disrupts iron homeostasis via LIF to confer oxaliplatin vulnerability in colorectal cancer. Biochim. Biophys. Acta BBA - Mol. Basis Dis. 1870 (6), 167210. 10.1016/j.bbadis.2024.167210 38704001

[B12] Khan S. U. Fatima K. Aisha S. Malik F. (2024). Unveiling the mechanisms and challenges of cancer drug resistance. Cell. Commun. Signal 22 (1), 109. 10.1186/s12964-023-01302-1 38347575 PMC10860306

[B13] Kuleshov M. V. Jones M. R. Rouillard A. D. Fernandez N. F. Duan Q. Wang Z. (2016). Enrichr: a comprehensive gene set enrichment analysis web server 2016 update. Nucleic Acids Res. 44 (W1), W90–W97. 10.1093/nar/gkw377 27141961 PMC4987924

[B14] Labrie M. Brugge J. S. Mills G. B. Zervantonakis I. K. (2022). Therapy resistance: opportunities created by adaptive responses to targeted therapies in cancer. Nat. Rev. Cancer 22 (6), 323–339. 10.1038/s41568-022-00454-5 35264777 PMC9149051

[B15] Laithy Y. E. Oliveira W. Pabba A. Qualizza A. Richard F. Athanasouli P. (2024). Chemotherapy-treated breast cancer cells activate the wnt signaling pathway to enter a diapause-DTP state [Preprint]. bioRxiv, 2024. 10.1101/2024.03.08.584051 PMC12809118 41117706

[B16] Liau B. B. Sievers C. Donohue L. K. Gillespie S. M. Flavahan W. A. Miller T. E. (2017). Adaptive chromatin remodeling drives glioblastoma stem cell plasticity and drug tolerance. Cell. Stem Cell. 20 (2), 233–246.e7. 10.1016/j.stem.2016.11.003 27989769 PMC5291795

[B17] Lin Y. Li C. Xiong W. Fan L. Pan H. Li Y. (2021). ARSD, a novel ERα downstream target gene, inhibits proliferation and migration of breast cancer cells via activating Hippo/YAP pathway. Cell. Death Dis. 12 (11), 1042. 10.1038/s41419-021-04338-8 34725332 PMC8560752

[B18] Livak K. J. Schmittgen T. D. (2001). Analysis of relative gene expression data using real-time quantitative PCR and the 2^−ΔΔCT^ method. Methods 25 (4), 402–408. 10.1006/meth.2001.1262 11846609

[B19] Marine J. C. Dawson S. J. Dawson M. A. (2020). Non-genetic mechanisms of therapeutic resistance in cancer. Nat. Rev. Cancer 20 (12), 743–756. 10.1038/s41568-020-00302-4 33033407

[B20] Mikubo M. Inoue Y. Liu G. Tsao M. S. (2021). Mechanism of drug tolerant persister cancer cells: the landscape and clinical implication for therapy. J. Thorac. Oncol. 16 (11), 1798–1809. 10.1016/j.jtho.2021.07.017 34352380

[B21] Mimura N. Fulciniti M. Gorgun G. Tai Y. T. Cirstea D. Santo L. (2012). Blockade of XBP1 splicing by inhibition of IRE1α is a promising therapeutic option in multiple myeloma. Blood 119 (24), 5772–5781. 10.1182/blood-2011-07-366633 22538852 PMC3382937

[B22] Ou Y. Wang S. J. Li D. Chu B. Gu W. (2016). Activation of SAT1 engages polyamine metabolism with p53-mediated ferroptotic responses. Proc. Natl. Acad. Sci. 113 (44), E6806–E6812. 10.1073/pnas.1607152113 27698118 PMC5098629

[B23] Pfeifer M. Brammeld J. S. Price S. Pilling J. Bhavsar D. Farcas A. (2024). Genome-wide CRISPR screens identify the YAP/TEAD axis as a driver of persister cells in EGFR mutant lung cancer. Commun. Biol. 7 (1), 497. 10.1038/s42003-024-06190-w 38658677 PMC11043391

[B24] R Core Team (2023). R: A language and Environment for Statistical Computing. Vienna, Austria: R Foundation for Statistical Computing. Available online at: https://www.r-project.org/ (Accessed April 13, 2026).

[B25] Rehman S. K. Haynes J. Collignon E. Brown K. R. Wang Y. Nixon A. M. L. (2021). Colorectal cancer cells enter a Diapause-like DTP state to survive chemotherapy. Cell. 184 (1), 226–242.e21. 10.1016/j.cell.2020.11.018 33417860 PMC8437243

[B26] Robin X. Turck N. Hainard A. Tiberti N. Lisacek F. Sanchez J. C. (2011). pROC: an open-source package for R and S+ to analyze and compare ROC curves. BMC Bioinforma. 12 (1), 77. 10.1186/1471-2105-12-77 21414208 PMC3068975

[B27] Roszkowska M. (2024). Multilevel mechanisms of cancer drug resistance. Int. J. Mol. Sci. 25 (22), 12402. 10.3390/ijms252212402 39596466 PMC11594576

[B28] Sharma S. V. Lee D. Y. Li B. Quinlan M. P. Takahashi F. Maheswaran S. (2010). A chromatin-mediated reversible drug-tolerant state in cancer cell subpopulations. Cell. 141 (1), 69–80. 10.1016/j.cell.2010.02.027 20371346 PMC2851638

[B29] Subramanian A. Narayan R. Corsello S. M. Peck D. D. Natoli T. E. Lu X. (2017). A next generation connectivity map: L1000 platform and the first 1,000,000 profiles. Cell. 171 (6), 1437–1452.e17. 10.1016/j.cell.2017.10.049 29195078 PMC5990023

[B30] Tam B. Y. Chiu K. Chung H. Bossard C. Nguyen J. D. Creger E. (2020). The CLK inhibitor SM08502 induces anti-tumor activity and reduces wnt pathway gene expression in gastrointestinal cancer models. Cancer Lett. 473, 186–197. 10.1016/j.canlet.2019.09.009 31560935

[B31] Tian Y. Wang X. Wu C. Qiao J. Jin H. Li H. (2024). A protracted war against cancer drug resistance. Cancer Cell. Int. 24 (1), 326. 10.1186/s12935-024-03510-2 39342202 PMC11439304

[B32] Wang X. Zhang H. Chen X. (2019). Drug resistance and combating drug resistance in cancer. Cancer Drug Resist 2 (2), 141–160. 10.20517/cdr.2019.10 34322663 PMC8315569

[B33] Wickham H. (2016). ggplot2: Elegant Graphics for Data Analysis. Cham: Springer International Publishing. 10.1007/978-3-319-24277-4

[B34] Wu T. Hu E. Xu S. Chen M. Guo P. Dai Z. (2021). clusterProfiler 4.0: a universal enrichment tool for interpreting omics data. Innovation 2 (3), 100141. 10.1016/j.xinn.2021.100141 34557778 PMC8454663

[B35] Yoo M. Shin J. Kim J. Ryall K. A. Lee K. Lee S. (2015). DSigDB: drug signatures database for gene set analysis. Bioinformatics 31 (18), 3069–3071. 10.1093/bioinformatics/btv313 25990557 PMC4668778

[B36] Zhou Q. Qi H. Zou Y. Yue Z. Gan J. Xu H. (2026). Hypoxia-induced XBP1s-MYDGF axis suppresses ferroptosis through UBQLN1-mediated stabilization of LCN2 in gastric cancer. Oncogene 45, 1786–1799. 10.1038/s41388-026-03760-6 41942632 PMC13139035

