# Supplementary material for: A recurrently perturbed 12-gene program marks drug-tolerant persister states across cancer types
Source: Front Bioinform. 2026 Aug 18;6:1882238. doi: 10.3389/fbinf.2026.1882238 (PMC13530430; doi:10.3389/fbinf.2026.1882238)
Supplement: Supplementary file 1 [file DataSheet1.docx]

Supplementary Material

A Recurrently Perturbed 12-Gene Program Marks Drug-Tolerant Persister States Across Cancer Types

**Supplementary Figures**

**Supplementary Figure S1.** Concordance-stratified analysis of PanDTP-12: Core versus Variable subsets. **(A)** Heatmap of gene-level log_2_FC across discovery datasets with row-side color indicating Core (≥3/4 concordance) or Variable (2/4 concordance) classification. **(B)** Paired lollipop plot of AUC values for Core_9 versus Full_12 signatures per dataset and **(C)** Forest plot of Cohen’s *d* effect sizes with 95% CI for Core_9 and Full_12 per dataset.

**Supplementary Figure S2. Senstivity analysis excluding GSE214537.** **(A)** Cleveland dot plot showing gene recurrence counts in the full 4-dataset analysis versus the 3-dataset drug-only analysis. All 12 PanDTP-12 genes remain recurrent at ≥3/3. **(B)** Split violin and swarm plot of module scores in the two remaining classifiable datasets after GSE214537 exclusion.

**Supplementary Figure S3.** Permutation null distributions for PanDTP-12 classification AUC. For each of the three classifiable discovery datasets, histograms show the distribution of AUC values from 5,000 random label permutations, with a KDE overlay and the observed AUC indicated by a dashed red line. Inset CDF plots show the cumulative density of null AUCs. The 95th percentile of each null distribution equals 1.0, reflecting the combinatorial constraints of very small sample sizes.

**Supplementary Figure S4.** Ferroptosis pathway enrichment in DTP models. **(A)** Lollipop plot of odds ratios from Fisher’s exact test for ferroptosis gene-set enrichment per discovery dataset (FerrDb v2 reference) and **(B)** Grouped dot plot of log_2_FC values for the four PanDTP-12 members overlapping with the ferroptosis gene set (SAT1, USP11, KLF5, XBP1) across all discovery datasets.

Supplementary Figure S5. RT-qPCR validation of RGS2 and PNP in INK128-induced DTP cells. Bar charts of relative expression (2^(−ΔΔCt), GAPDH-normalised) of RGS2 and PNP in MCF-7 (blue) and HCT116 (red) DTP versus control cells; mean ± SD of three biological replicates. Dashed line at fold change = 1.0. Significance from one-sided t-tests: *p < 0.05, **p < 0.01; ns, not significant. Both genes are significantly upregulated in HCT116 (RGS2 5.28-fold, p = 0.013; PNP 9.45-fold, p = 0.002) but non-significantly downregulated in MCF-7 (RGS2 0.55-fold; PNP 0.72-fold), indicating cell-line-context-dependent directionality.

Supplementary Figure S6. Cross-dataset PCA and correlation clustering of the four discovery datasets. All 54 samples were quantile-normalised, log2-transformed and reduced to the 1,000 most variable genes to enable comparison across series processed on different scales (GSE153183 and GSE214537 log-scaled; GSE189764 and GSE246690 linear). (A) PCA; points coloured by dataset, shaped by condition (control, persister, or unlabelled for GSE246690 where sample-level condition was not annotated); stars mark dataset centroids (PC1 = 26.1%, PC2 = 18.5%). (B) Hierarchical clustering of samples on 1 − Spearman correlation; the coloured bar marks each leaf's dataset. GSE214537 (fasting) is not an outlier: it correlates positively with all three pharmacological models (Spearman r = 0.56–0.68, highest with the pancreatic GSE189764, r = 0.68) and clusters among them, whereas the lung osimertinib dataset (GSE153183) is the most distinct series.

**Supplementary Tables**

**Supplementary Table S1.** Gene-level concordance with log2FC per dataset, majority direction, concordance score, and Core/Variable classification for all 12 PanDTP-12 genes.

| **Gene** | **GSE153183_log2FC** | **GSE214537_log2FC** | **GSE189764_log2FC** | **GSE246690_log2FC** | **Majority_Direction** | **Concordance** | **Classification** |
| --- | --- | --- | --- | --- | --- | --- | --- |
| SAT1 | 0.82 | 3.19 | 2.1 | 2.36 | up | 04-Apr | Core |
| CLK1 | 0.8 | 2.78 | 1.48 | 1.12 | up | 04-Apr | Core |
| ARSD | 0.96 | 1.04 | 1.49 | 1.75 | up | 04-Apr | Core |
| USP11 | 1 | 1.02 | 1.62 | 0.88 | up | 04-Apr | Core |
| SKIL | -0.94 | 1.51 | 1.15 | 0.89 | up | 03-Apr | Core |
| MXI1 | -1.93 | 1.04 | 0.96 | 1.33 | up | 03-Apr | Core |
| XBP1 | 0.88 | 1.86 | 1.21 | -0.83 | up | 03-Apr | Core |
| RGS2 | 1.06 | 3.46 | 2.37 | -1.74 | up | 03-Apr | Core |
| KLF5 | -3.49 | 1.4 | 2.75 | 2.16 | up | 03-Apr | Core |
| PBX1 | 1.36 | -2.06 | -2.81 | 2.41 | mixed | 02-Apr | Variable |
| DNAJB4 | -1.24 | 3.44 | 1.21 | -0.91 | mixed | 02-Apr | Variable |
| PNP | -1.09 | 1.22 | 1.25 | -1.88 | mixed | 02-Apr | Variable |

**Supplementary Table S2.** Two panels: (A) gene recurrence showing all 12 survive at 3/3 after GSE214537 exclusion; (B) classification AUC in remaining datasets.

| Panel A: Gene Recurrence After Excluding GSE214537 | | | | |  |
| --- | --- | --- | --- | --- | --- |
| Gene | Recurrence_4datasets | Recurrence_3datasets | Survives | Concordance | Classification |
| ARSD | 04-Apr | 03-Mar | Yes | 04-Apr | Core |
| CLK1 | 04-Apr | 03-Mar | Yes | 04-Apr | Core |
| DNAJB4 | 04-Apr | 03-Mar | Yes | 02-Apr | Variable |
| KLF5 | 04-Apr | 03-Mar | Yes | 03-Apr | Core |
| MXI1 | 04-Apr | 03-Mar | Yes | 03-Apr | Core |
| PBX1 | 04-Apr | 03-Mar | Yes | 02-Apr | Variable |
| PNP | 04-Apr | 03-Mar | Yes | 02-Apr | Variable |
| RGS2 | 04-Apr | 03-Mar | Yes | 03-Apr | Core |
| SAT1 | 04-Apr | 03-Mar | Yes | 04-Apr | Core |
| SKIL | 04-Apr | 03-Mar | Yes | 03-Apr | Core |
| USP11 | 04-Apr | 03-Mar | Yes | 04-Apr | Core |
| XBP1 | 04-Apr | 03-Mar | Yes | 03-Apr | Core |
| Panel B: Classification Performance Without GSE214537 | | | | | |
| Dataset | n_genes | AUC | Strict_Separation | Mean_Control | Mean_DTP |
| GSE153183 | 12 | 1 | Yes | -0.7007 | 0.7007 |
| GSE189764 | 12 | 1 | Yes | -0.7512 | 0.7512 |

**Supplementary Table S3.** Permutation null distributions (5,000 permutations per dataset, observed AUC, null mean/SD/95th/99th percentile, permutation p).

| Dataset | Observed_AUC | Null_Mean | Null_SD | Null_95th_Percentile | Null_99th_Percentile | Null_Max | Permutation_p | N_permutations |
| --- | --- | --- | --- | --- | --- | --- | --- | --- |
| GSE153183 | 1 | 0.4942 | 0.3225 | 1 | 1 | 1 | 0.1638 | 5000 |
| GSE189764 | 1 | 0.5054 | 0.2552 | 1 | 1 | 1 | 0.0516 | 5000 |
| GSE214537 | 1 | 0.5019 | 0.2552 | 1 | 1 | 1 | 0.051 | 5000 |

**Supplementary Table S4.** Ferroptosis enrichment per dataset (odds ratio, Fisher's p, up/down counts, PanDTP-12 ferroptosis member.

| Dataset | Cancer_Type | Total_DEGs | Ferroptosis_DEGs | Ferroptosis_Background | Up | Down | Odds_Ratio | Fisher_p | PanDTP12_Ferroptosis_Members |
| --- | --- | --- | --- | --- | --- | --- | --- | --- | --- |
| GSE153183 | Lung (NSCLC) | 1578 | 3 | 46 | 3 | 0 | 0.72 | 0.7862 | SAT1, USP11, KLF5, XBP1 |
| GSE189764 | Pancreatic | 3889 | 13 | 44 | 11 | 2 | 3.26 | 0.0009 | SAT1, USP11, KLF5, XBP1 |
| GSE214537 | Colorectal | 1233 | 13 | 46 | 13 | 0 | 0.72 | 0.8732 | SAT1, USP11, KLF5, XBP1 |
| GSE246690 | Melanoma | 4489 | 15 | 46 | 2 | 13 | 6.08 | <0.0001 | SAT1, USP11, KLF5, XBP1 |

**Supplementary Table S5.** Two-sided p-values for RT-qPCR validation of 10 PanDTP-12 genes in MCF-7 and HCT116 DTP cells. Values computed from delta-Ct two-sided unpaired Student's t-tests (n = 3 biological replicates per group). MCF-7: 3/10 significant (ARSD, PBX1, USP11); HCT116: 10/10 significant.

| Gene | MCF7_log2FC | MCF7_FC | MCF7_p_onesided | MCF7_p_twosided | MCF7_sig_1s | MCF7_sig_2s | HCT116_log2FC | HCT116_FC | HCT116_p_onesided | HCT116_p_twosided | HCT116_sig_1s | HCT116_sig_2s |
| --- | --- | --- | --- | --- | --- | --- | --- | --- | --- | --- | --- | --- |
| ARSD | 3.42 | 10.7 | 0.0016 | 0.0031 | ** | ** | 1.89 | 3.7 | 0.0127 | 0.0254 | * | * |
| CLK1 | 1.81 | 3.5 | 0.0528 | 0.1057 | ns | ns | 3.15 | 8.9 | 0.0018 | 0.0037 | ** | ** |
| DNAJB4 | 1.45 | 2.7 | 0.0653 | 0.1306 | ns | ns | 4.62 | 24.6 | 0.0013 | 0.0026 | ** | ** |
| KLF5 | 1.47 | 2.8 | 0.0369 | 0.0737 | * | ns | 2.21 | 4.6 | 0.0114 | 0.0227 | * | * |
| MXI1 | 1.98 | 3.9 | 0.0368 | 0.0737 | * | ns | 5.45 | 43.8 | 0.0006 | 0.0012 | ** | ** |
| PBX1 | 2.46 | 5.5 | 0.0185 | 0.0369 | * | * | 2.86 | 7.2 | 0.0143 | 0.0285 | * | * |
| SAT1 | 0.66 | 1.6 | 0.263 | 0.526 | ns | ns | 3.72 | 13.2 | 0.0004 | 0.0008 | *** | *** |
| SKIL | 1.7 | 3.3 | 0.0676 | 0.1352 | ns | ns | 7.11 | 138.4 | 0.0007 | 0.0015 | ** | ** |
| USP11 | 2.97 | 7.8 | 0.0114 | 0.0229 | * | * | 4.44 | 21.7 | 0.0008 | 0.0016 | ** | ** |
| XBP1 | 2.02 | 4.1 | 0.0497 | 0.0993 | * | ns | 2.68 | 6.4 | 0.0008 | 0.0016 | ** | ** |

Supplementary Table S6. RT-qPCR validation of RGS2 and PNP in MCF-7 and HCT116 DTP cells. Per-gene log2FC, linear fold change (2^(−ΔΔCt), GAPDH-normalised), and one- and two-sided unpaired Student's t-test p-values (n = 3 biological replicates per group); ↓ indicates downregulation. (A) Summary statistics. (B) Raw Ct values (per replicate). Both genes are significantly upregulated in HCT116 and non-significantly downregulated in MCF-7.

| **Gene** | **MCF7_log2FC** | **MCF7_FC** | **MCF7_p_1s** | **MCF7_p_2s** | **MCF7_sig** | **HCT116_log2FC** | **HCT116_FC** | **HCT116_p_1s** | **HCT116_p_2s** | **HCT116_sig** |
| --- | --- | --- | --- | --- | --- | --- | --- | --- | --- | --- |
| RGS2 | -0.88 | 0.55 | 0.7853 | 0.4293 | ns (↓) | 2.40 | 5.28 | 0.0129 | 0.0259 | * |
| PNP | -0.48 | 0.72 | 0.6855 | 0.6291 | ns (↓) | 3.24 | 9.45 | 0.0018 | 0.0035 | ** |

(B) Raw Ct values (per replicate; GAPDH = reference gene).

| **Cell line** | **Gene** | **Control Ct (rep 1, 2, 3)** | **DTP Ct (rep 1, 2, 3)** |
| --- | --- | --- | --- |
| HCT116 | GAPDH | 18.54, 18.82, 18.30 | 19.41, 18.04, 19.18 |
| HCT116 | RGS2 | 22.61, 23.85, 22.05 | 20.64, 21.07, 20.57 |
| HCT116 | PNP | 23.04, 23.55, 23.11 | 20.22, 20.50, 20.22 |
| MCF-7 | GAPDH | 21.04, 22.26, 20.11 | 21.24, 21.77, 20.08 |
| MCF-7 | RGS2 | 22.08, 21.41, 22.23 | 22.26, 23.07, 22.70 |
| MCF-7 | PNP | 23.03, 22.85, 23.32 | 23.56, 23.34, 23.40 |
